# CAMKK2 regulates mitochondrial function by controlling succinate dehydrogenase expression, post-translational modification, megacomplex assembly, and activity in a cell-type-specific manner

**DOI:** 10.1186/s12964-021-00778-z

**Published:** 2021-09-25

**Authors:** Mohammad Golam Sabbir, Carla G. Taylor, Peter Zahradka

**Affiliations:** 1 Canadian Centre for Agri-Food Research in Health and Medicine, St. Boniface Albrechtsen Research Centre, Room R2034 - 351 Taché Avenue, Winnipeg, MB R2H 2A6 Canada; 2 Alzo Biosciences Inc., San Diego, CA USA; 3 Department of Food and Human Nutritional Sciences, University of Manitoba, Winnipeg, MB R3T 2N2 Canada; 4 Department of Physiology and Pathophysiology, University of Manitoba, Winnipeg, MB R3E 0J9 Canada

**Keywords:** CAMKK2, Succinate dehydrogenase, Oxidative phosphorylation, Respiratory supercomplex, Respiration

## Abstract

**Background:**

The calcium (Ca2+)/calmodulin (CAM)-activated kinase kinase 2 (CAMKK2)-signaling regulates several physiological processes, for example, glucose metabolism and energy homeostasis, underlying the pathogenesis of metabolic diseases. CAMKK2 exerts its biological function through several downstream kinases, therefore, it is expected that depending on the cell-type-specific kinome profile, the metabolic effects of CAMKK2 and its underlying mechanism may differ. Identification of the cell-type-specific differences in CAMKK2-mediated glucose metabolism will lead to unravelling the organ/tissue-specific role of CAMKK2 in energy metabolism. Therefore, the objective of this study was to understand the cell-type-specific regulation of glucose metabolism, specifically, respiration under CAMKK2 deleted conditions in transformed human embryonic kidney-derived HEK293 and hepatoma-derived HepG2 cells.

**Methods:**

Cellular respiration was measured in terms of oxygen consumption rate (OCR). OCR and succinate dehydrogenase (SDH) enzyme activity were measured following the addition of substrates. In addition, transcription and proteomic and analyses of the electron transport system (ETS)-associated proteins, including mitochondrial SDH protein complex (complex-II: CII) subunits, specifically SDH subunit B (SDHB), were performed using standard molecular biology techniques. The metabolic effect of the altered SDHB protein content in the mitochondria was further evaluated by cell-type-specific knockdown or overexpression of SDHB.

**Results:**

CAMKK2 deletion suppressed cellular respiration in both cell types, shifting metabolic phenotype to aerobic glycolysis causing the Warburg effect. However, isolated mitochondria exhibited a cell-type-specific enhancement or dampening of the respiratory kinetics under CAMKK2 deletion conditions. This was mediated in part by the cell-type-specific effect of CAMKK2 loss-of-function on transcription, translation, post-translational modification (PTM), and megacomplex assembly of nuclear-encoded mitochondrial SDH enzyme complex subunits, specifically SDHB. The cell-type-specific increase or decrease in SDHs protein levels, specifically SDHB, under CAMKK2 deletion condition resulted in an increased or decreased enzymatic activity and CII-mediated respiration. This metabolic phenotype was reversed by cell-type-specific knockdown or overexpression of SDHB in respective CAMKK2 deleted cell types. CAMKK2 loss-of-function also affected the overall assembly of mitochondrial supercomplex involving ETS-associated proteins in a cell-type-specific manner, which correlated with differences in mitochondrial bioenergetics.

**Conclusion:**

This study provided novel insight into CAMKK2-mediated cell-type-specific differential regulation of mitochondrial function, facilitated by the differential expression, PTMs, and assembly of SDHs into megacomplex structures.
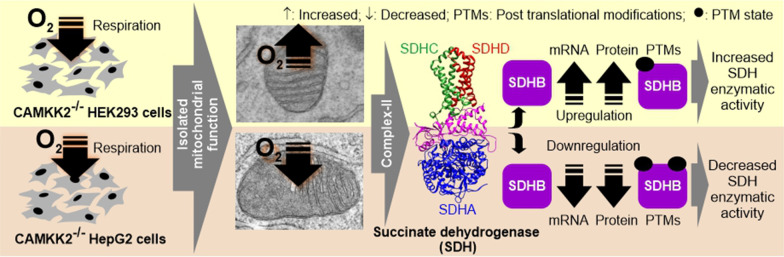

**Video Abstract**

**Supplementary Information:**

The online version contains supplementary material available at 10.1186/s12964-021-00778-z.

## Background

CAMKK2, a Serine/Threonine protein kinase, plays important role in a variety of physiological processes including glucose homeostasis and utilization, adipogenesis, as well as whole-body energy balance [1, 2]. Activated CAMKK2 directly phosphorylates multiple downstream effectors including Ca^2+^/CAM-dependent protein kinase I and 4 (CAMKI and CAMK4), AMP-activated protein kinase (AMPK), and the NAD-dependent protein deacetylase—Sirtuin 1 (SIRT1), each have their distinct signaling effects on cellular metabolism [2]. For example, AMPK is a cellular energy sensor activated by metabolic stresses that inhibits mitochondrial ATP production or accelerates ATP consumption [3, 4]. Activated AMPK stimulates glucose uptake and lipid oxidation to produce energy while turning off energy-consuming processes including glucose and lipid production to restore energy balance and maintain homeostasis [5, 6]. On the other hand, the CAMK4 signaling cascade activates the cAMP response element-binding (CREB) transcription factor regulating the expression of genes associated with cellular metabolism and growth [2]. For example, CREB upregulates the expression of glucose transporters thereby increasing the uptake of glucose and elevating intracellular glucose levels [7, 8]. CREB also regulates glycolytic enzymes [7, 9] and enables mitochondrial respiration and biogenesis under stress conditions [10]. CAMK4 inhibition results in decreased levels of glycolytic intermediates suggesting a direct role in glucose metabolism [11]. The CAMKK2 downstream effector SIRT1 [12] is also a key modulator of hepatic gluconeogenesis [13] and glucose homeostasis [14]. Thus, multiple mechanisms exist that may link disturbed calcium-CAMKK2 signaling with altered cellular metabolism underlying the pathogenesis of diseases. The mechanistic basis of CAMKK2-mediated glucose oxidation and mitochondrial bioenergetics is not well characterized despite a wealth of knowledge accumulated over the years regarding CAMKK2-downstream effector-mediated regulation of cellular glucose homeostasis and the maintenance of energy balance.

Energy homeostasis is a complex biological process involving the coordinated regulation of food intake, energy production, and expenditure. It has been suggested that CAMKK2 regulates whole-body energy balance by coordinating the actions of key metabolic tissues [2]. CAMKK2 is expressed in a variety of cell types in different tissues [15–17]. Different cells within an organ or tissue environment have different metabolic roles and energy demands. Besides, it is a well-established fact that the proteome, including the kinome, differs between the constituent cell types in any organ or tissue system since it is meant to meet the specific niche of structural and functional goals. In this context, it is important to note that both RNA sequencing as well as validated antibody-based immunohistochemistry studies under the Human Protein Atlas (HPA) project [18] revealed that CAMKK2 is expressed in diverse human tissues and cell types including many transformed cells. Our previous studies revealed the presence of CAMKK2 full-length mRNA in adult human adipose, artery, bone marrow, cortex, cerebellum, intestine, liver, skeletal muscle, and skin tissues [17]. Furthermore, we characterized CAMKK2 expression in primary human endothelial and myeloid cells [17], and our previous studies demonstrated CAMKK2 expression in different transformed human cell types including human embryonic kidney-derived HEK293 cells, hepatoma-derived HepG2 cells [16, 17], and a hybrid (endothelial and alveolar) cell line with endothelial characteristics, designated EA.hy926 [17]. Thus, the expression of CAMKK2 in diverse cell types leads to an important question—does CAMKK2 function uniformly in regulating cell-type-specific glucose metabolism? This important biological question was addressed in this study by performing a comparative bioenergetics analysis of cellular glucose metabolism and mitochondrial respiration using multiple CAMKK2 deleted HEK293 and HepG2 cell clones. Our choice of these two CAMKK2 expressing cell lines was influenced by their distinctive metabolic phenotypes which are reflected in the differences in their proteome composition [19], physiological properties [20], metabolite signature [21], and cell doubling time (HEK293 vs HepG2: ~ 24 h vs ~ 44 h) [22–24]. In addition, the expression of oxidative phosphorylation (OXPHOS)-associated proteins, specifically SDH enzyme complex subunits, and their PTMs and association into multiprotein complexes, were studied to correlate with cell-type-specific differences in mitochondria bioenergetics. Furthermore, the mitochondrial ultrastructure was compared between the cell types. Overall our findings indicate that CAMKK2-mediated cellular glucose metabolism is regulated in a cell-type-specific manner at multiple levels, including gene expression, protein modification, and protein complex assembly.

## Methods

### CAMKK2 deleted HEK293 and HepG2 cell clones

The HEK293 cell line was originally developed by transformation of primary cultures of human embryonic kidney (HEK) cells with sheared adenovirus 5 DNA [25]. The HepG2 cells were derived from primary liver carcinomas [26]. The CRISPR/Cas9-mediated CAMKK2 deleted (CAMKK2^−/−^) HEK293 and HepG2 cell clones were generated as previously described [16]. The cells were cultivated in Dulbecco’s modified Eagle’s medium (DMEM) supplemented with 10% heat-inactivated FBS and 1 × antibiotic antimycotic solution (Sigma, Cat No: A5955).

### Mitochondrial function test

Oxygen consumption rate (OCR) and extracellular acidification rates (ECAR) were measured simultaneously using a Seahorse Biosciences XF24 analyzer (Agilent) [17, 27]. Cultured cells were grown in 24 well assay plates overnight. Cells were then washed thrice in a pre-warmed XF assay medium (non-buffered DMEM) supplemented with 1 mM sodium pyruvate (pH 7.4) and finally, 475 µl assay medium was added to each well and incubated in a non-CO_2_ incubator for 1 h. Meanwhile, appropriate volumes of pre-warmed glucose, oligomycin, 2-[2-[4-(trifluoromethoxy)phenyl]hydrazinylidene]-propanedinitrile (FCCP), rotenone and antimycin A were added into injector ports A, B, C, and D of the sensor cartridge, respectively. The final concentrations of injections were as follows: 5 mM Glucose (Glu), 2 µM oligomycin (Oligo), 1 µM FCCP, and 0.5 µM rotenone/antimycin-A (Rtn/AA). The cartridge was calibrated by the XF24 analyzer (Agilent Seahorse Bioscience, Billerica, MA, USA), and the OCR was measured using the Agilent XF Cell mitochondrial function test according to the manufacturer's instructions. Briefly, basal OCRs were measured in the absence of glucose followed by the sequential addition to each well of glucose, oligomycin, FCCP, and rotenone/antimycin-A. The OCR values were normalized by setting the pre-glucose injection OCR as 100%.

### Isolation and enrichment of endoplasmic reticulum (ER)/mitochondrial fraction

Mitochondria were isolated by a method previously described [17, 27]. Briefly, 80–90% confluent cells were washed with 1 × phosphate-buffered saline (PBS) and harvested in mitochondrial stabilization buffer (MSB) containing 70 mM sucrose, 210 mM mannitol, 5 mM HEPES pH 7.2, 1 mM EGTA, 5 mM MgCl_2_, 10 mM KH_2_PO_4_, and pH adjusted to 7.4. The cells were disrupted with a Teflon Dounce homogenizer and the homogenate was centrifuged at 800 g for 10 min at 4 °C. Following centrifugation, the supernatant was decanted through 2 layers of cheesecloth into a separate tube and centrifuged at 8000 g for 10 min at 4 °C. After removal of the supernatant, the pellet was resuspended in mitochondrial isolation buffer, washed thoroughly and the centrifugation was repeated. The final pellet was resuspended in lysis buffer and used for immunoblotting. The enriched mitochondrial pellet was also used for transmission electron microscope-based examination as well as bioenergetics analysis for ETS function. The entire process of mitochondrial enrichment was performed either at 4 °C or by keeping the intermediate/enriched fractions on ice to minimize any hypoxia-induced effect.

### Transmission electron microscopy (TEM)

TEM was performed as described previously [17, 27]. Briefly, the cells were grown on nitrocellulose membranes and subsequently fixed with 2% glutaraldehyde in Sorenson’s buffer (133 mM Na_2_HPO_4_/KH_2_PO_4_, pH 7.4) at 4 °C for 2 h. The enriched ER/mitochondrial pellet resuspended in MSB buffer was layered on top of a circular piece of nitrocellulose membrane in a 96 well plate and centrifuged at 4000*g* for 30 min using a horizontal plate rotor and subsequently fixed with 2% glutaraldehyde in Sorenson’s buffer at 4 °C for 2 h. Following fixation, the membranes containing a monolayer of cells or ER/mitochondrial pellets were dehydrated and finally embedded in epoxy resin and polymerized at 60 °C overnight. Sections were cut on a Leica EM UC7 ultra-microtome. Semi-thin sections (0.5 µm) were stained with toluidine blue and examined under light microscopy to identify the area of interest and confirm the orientation of the cells. Ultrathin sections (70 nm) were then transferred to copper grids (Ted Pella Inc), and stained with uranyl acetate and lead citrate, and examined on a Phillips CM 100 Compustage transmission electron microscope. Digital micrographs were captured with an AMT CCD camera (Deben).

### Western blotting and quantification

Relative quantification of proteins by Western blot analysis was performed as described previously [17]. Briefly, 20–30 µg of protein was loaded for each sample to run two SDS-PAGE-based gels in parallel; one of them was used for immunoblotting and the other was used for oriole staining and subsequent imaging using the ChemiDoc MP Imaging System (Bio-Rad). The 1–1.5 mm thick polyacrylamide gels were operated in a vertical electrophoresis chamber at a field strength of 10–20 V/cm, respectively. A reference protein detected in the same immunoblot as the target protein-of-interest was used for normalization during the relative quantification of proteins-of-interest. The choice of reference protein was decided based on the uniformity of the reference protein level under all experimental conditions as determined by visual inspection of the image-J-based plot profile of the immunoblot band intensities. The protein-of-interest bands within a single immunoblot were first normalized by dividing the band intensities with the intensities of the corresponding reference protein bands [28]. Next, the normalized band intensity of the protein-of-interest in the control (X) was converted to 100% by using the formulae (X/X*100). The protein-of-interest bands in the experimental group within the same immunoblot were then converted to a percent of control by using the formula (Y/X)*100, where “Y” is the normalized band intensity of the protein-of-interest in the experimental set. This allows comparison between different immunoblots derived from independent experiments or biological replicates. Table 1 lists the primary antibodies used in this study.

**Table 1:** List of reagents

Name	Source	Type	Host species	Cat. No	Lot No	Dilution
*Antibodies*						
α-tubulin (TU-02)	SCBT	Monoclonal	Mouse	Sc-8035	L1416	1:1000
CAMKK2(ZZ9)	SCBT	Monoclonal	Mouse	Sc-100364	A0220	1:1000
GAPDH	SCBT	Monoclonal	Mouse	Sc-25778	C0910	1:1000
OXPHOS-cocktail	Abcam	Monoclonal	Mouse	MS601	P9552	1:2000
NDUFB8 (CI)	Abcam	Monoclonal	Mouse	ab110242	NA	NA
SDHB (CII)	Abcam	Monoclonal	Mouse	ab14714	NA	NA
UQCRC2 (CIII)	Abcam	Monoclonal	Mouse	ab14745	NA	NA
COXII (CIV)	Abcam	Monoclonal	Mouse	ab110258	NA	NA
ATP5A (CV)	Abcam	Monoclonal	Mouse	ab14748	NA	NA
SDHA	Abcam	Monoclonal	Rabbit	AB137040	GR3252256-5	1:1000
SDHA	SCBT	Monoclonal	Mouse	Sc390381	E2720	1:1000
SDHB	SCBT	Monoclonal	Mouse	Sc-271548	L2619	1:1000
SDHC	SCBT	Monoclonal	Mouse	SC-515102	F0719	1:1000
VDAC1(B-6)	SCBT	Monoclonal	Mouse	Sc-390996	D1918	1:1000

Gene	Forward	Reverse	Amplicon (base pair)
*Multiplex/qRT-PCR primers*			
SDHA	CAGTCAAGGCGAAAGGTTTATG	CCCAGCGTTTGGTTTAATTGG	508
SDHB	TCCGAAGATCATGCAGAGAAG	TACAGCAGGCACAGAGAATG	312
SDHC	CCCAGCATCATCTTCCTACAC	TGCAGCCACCTCATCTTTAG	202
SDHC*	GTGGCACTGGTATTGCTTTG	CACAGAGCTGGCATTGTTTC	488
SDHD	AGCTCTGTTGCTTCGAACTC	CAGATGCCCACATCGTGATAG	399

*Dicer-substrate short interfering RNAs (DsiRNAs): sense and antisense*		
SDHB	GUAUUGGAUGCUUUAAUCAAGAUTA	UAAUCUUGAUUAAAGCAUCCAAUACCA
Control	CCUUCCUCUCUUUCUCUCCCUUGUG	CACAAGGGAGAGAAAGAGAGGAAGG

The cell lysates were prepared in 1 × RIPA lysis and extraction buffer (ThermoFisher Scientific, Cat No: 89900) supplemented with 1 × Halt protease and phosphatase inhibitor cocktail (ThermoFisher Scientific, Cat No: 78441). The protein lysates were denatured in Laemmli buffer containing 2% SDS, 10% glycerol, 0.002% bromophenol blue, and 0.75 M Tris–HCl pH 6.8 supplemented with 100 mM DTT. The ER/mitochondrial lysates that were meant for anti-OXPHOS and anti-SDH antibody-based quantification were heat-denatured at 55 °C for 10 min. The lysates for the rest of the antibodies were boiled in a water bath for 10 min. The SDS-PAGE separated proteins were transferred to 0.2 μm nitrocellulose membrane using a Trans-Blot Turbo Transfer System (Bio-Rad). The membranes were blocked using EveryBlot Blocking Buffer (Bio-Rad, Cat No: 12010947). The immunoblots were detected using chemiluminescence, imaged with the ChemiDoc MP Imaging System (Bio-Rad), and quantified using ImageJ (version 1.48) Software [29].

### Isolated mitochondrial function test

We performed “coupling” and “electron flow” assays using microgram (10 µg) quantities of enriched mitochondria derived from parental (wild-type: WT) and CAMKK2 deleted HEK293 and HepG2 cells (only electron flow assay) and different respiratory complex inhibitors to study ETS function [30] (Additional file 1: Fig. S1). To minimize variability between wells, 10 µg of enriched mitochondria was first diluted in 50 µL of MSB supplemented with 0.5% fatty acid-free BSA (pH 7.2), known as assay buffer, and delivered to each well of a Seahorse XF24 analyzer (Agilent) and spun in a swinging bucket rotor centrifuge at 2000 g for 20 min at 4 °C. After centrifugation, 450 µL of assay buffer and appropriate substrate were added to each well and incubated at 37 °C for 10 min and the experiments were initiated immediately. For the electron flow assay, 10 mM pyruvate (Pyr), 2 mM malate (Mal), and 4 µM FCCP were used at the beginning, and 2 µM rotenone (Rtn), 10 mM succinate (Succ), 4 µM antimycin A (AA), 10 mM ascorbate + 100 µM N,N,N9,N9-Tetramethylp-phenylenediamine (Asc + TMPD) were sequentially injected, and measurements of OCR were taken after each injection. It is important to note that in both the coupling and electron flow assays, appropriate and uniform loading of the mitochondrial sample is a critical factor for OCR measurement and comparison. Overloading may deplete O_2_ from the microchamber (zero O_2_ tension) during the measurement period and the system may not have an adequate time to recover to normoxia (return to ambient O_2_ tension, 158 mmHg) before the next set of measurements which may lead to an erroneous result [30]. Therefore, in both assays, thorough mixing of the assay media was performed between each measurement. The duration of typical mixing and measurement cycles was adopted from the protocol standardized by Rogers et al. (Additional file 8: Table S1) [30].

### Blue-native polyacrylamide gel electrophoresis (BN-PAGE)

The BN-PAGE analysis was performed as described previously [27]. Briefly, the lysates were prepared by sonicating the proteins in 1 × BN-PAGE lysis buffer (pH 7) containing 20 mM Bis–Tris, 500 mM 6-aminocaproic acid (Sigma, Cat. No: A2504), 20 mM NaCl, 2 mM EDTA, 10% glycerol, 1.5% *n*-Dodecyl β-D-maltoside (Sigma, Cat. No: D4641), and supplemented with 1 × Halt protease and phosphatase inhibitor cocktail (ThermoFisher Scientific, Cat. No: 1861281). The proteins and multiprotein complexes (MPCs) were then separated under native conditions in a 4–15% gradient 1.0 mm thick and 13.3 × 8.7 cm BN-PAGE gel using a cathode buffer (pH 7) containing 15 mM Bis–Tris, 50 mM Tricine, and 0.002% Coomassie blue G250, and an anode buffer (pH 7) containing 50 mM Bis–Tris. The first dimension BN-PAGE gel was electrophoresed at 100 V in a cold room (4 °C) until the samples traversed the 3.2% stacking gel and entered the separating gel. Subsequently, the voltage was increased to 180 V and allowed to run until the dye front reached the end of the gel. After a run, the gel strips (individual lanes) were carefully excised including the 3.2% stacking gel and immersed in freshly prepared sample buffer containing 12.5 mM Tris–HCl (pH 6.8), 4% SDS, 20% glycerol, 100 mM DTT, and 0.02% bromophenol blue, for 30 min at 50 °C. Subsequently, the proteins in the gel slices were separated in the second dimension using SDS-PAGE and immunoblotted. As a result, the component monomeric proteins in the MPCs would appear on a vertical line in the second dimension corresponding to the MPCs separated in the first dimension.

### RNA extraction, cDNA synthesis, multiplex reverse-transcription polymerase chain reaction (RT-PCR) and quantitative real time-PCR (qRT-PCR)

Total RNA from HEK293 and HepG2 cells were extracted with Trizol reagent (ThermoFisher Scientific, Cat. No: 15596026) as per the manufacturer’s recommended protocol. Total RNA (1 µg) was treated with RNase-free DNase I (New England Biolabs Inc., Cat. No: M0303) at 37 °C for 15 min, subsequently heat-inactivated at 75 °C for 10 min and used for cDNA synthesis. The first-strand cDNA was synthesized using an iScript cDNA synthesis kit (Bio-Rad, Cat. No: 1708891). Multiplex RT-PCR was performed as described previously [17]. Briefly, a 50 µL reaction mix containing 1 × buffer, 2 mM dNTP mix, 0.2 µM oligonucleotide primers, cDNAs equivalent to 100 ng total RNA, and 1.25 units DreamTaq™ Hot Start DNA Polymerase (ThermoFisher Scientific, Cat. No: EP1701), respectively, was prepared and amplified using 98/95 °C for 1 min, 35 cycles of 95 °C for 10 s, 62 °C for 10 s and 72 °C for 30 s. The RT-PCR products were separated using agarose gel electrophoresis and visualized. The qPCR was performed in a Mastercycler®ep realplex real-time PCR system (Eppendorf, Hamburg, Germany) using SYBR green dye (ThermoFisher Scientific, Cat. No: S7563) and cDNAs equivalent to 100 ng total RNA. The PCR efficiencies were calculated using the formula *E* = 10^[−1/slope]^. The efficiency of amplification was checked for all targets by performing a series of serial dilutions of the template for each primer pair in triplicate [31]. The calculated PCR efficiency for all target genes was between 98%-99%. The relative gene expression was calculated using 2 − ΔΔCT method [32].

### Isoelectric focusing (IEF)

Isoelectric focusing was performed as previously described [17]. Briefly, 50 µg of total cell lysate was precipitated by acetone and dissolved in rehydration buffer containing 8 M Urea, 2% CHAPS, 50 mM dithiothreitol (DTT) and 0.2% Bio-Lyte ampholytes pH 3-10. The dissolved proteins were then incubated in BioRad readystrip IPG strips pH 3-10 nonlinear (NL) 11 cm strips (ThermoFisher Scientific) overnight and focused at 175 V for 15 min, 175–8000 V ramp for 1 h, and 8000 V for 30,000 V-hours. After focusing, the proteins in the strips were reduced (by DTT), alkylated (by iodoacetamide), and resolved on 2D SDS-PAGE and immunoblotted.

### Knockdown and overexpression of SDHB

Chemically synthesized Dicer-Substrate Short Interfering RNA (DsiRNA) [33] targeted to exon 3 was used for knockdown of the SDHB gene. The control and SDHB-targeted DsiRNAs (Table 1) were transfected in CAMKK2^−/−^ HEK293 cells using Lipofectamine™ MessengerMAX™ Transfection Reagent (ThermoFisher Scientific, Cat. No: LMRNA001). The transfected cells were cultured for 48 h and then harvested for ER/mitochondrial enrichment, SDH enzyme activity measurement, and Western blotting. The full-length SDHB open reading frame (ORF: Accession number: NM_0030000.3) was chemically synthesized in the pcDNA3.1(+)N-DYK vector (GenScript: clone identification number: OHu18105C) and transfected in CAMKK2^−/−^ HepG2 cells using Lipofectamine™ 3000 Transfection Reagent (ThermoFisher Scientific, Cat. No: L3000001). The transfected cells were selected by adding 800 μg/mL G-418 (Sigma, Cat. NO: 4727878001) to the cell culture medium, and a pool of SDHB overexpressed cell populations were harvested after two weeks of culture. Subsequently, the SDHB overexpressed cells were expanded and used for ER/mitochondrial enrichment, SDH enzyme activity measurement, and Western blotting.

### Colorimetric SDH enzyme activity assay

The SDH enzymatic activity was measured by using a kit (Sigma, Cat. No: MAK197) according to the manufacturer’s protocol [34]. Briefly, the enriched ER/mitochondrial fractions from parental and CAMKK2^−/−^ ± SDHB knockdown/overexpressed cell types were lysed under ice-cold conditions using the lysis buffer provided in the kit, supplemented with 1 × Halt protease and phosphatase inhibitor cocktail (ThermoFisher Scientific, Cat No: 78441). Equal amounts of protein were used for the enzymatic activity assay. The SDH activity was determined by the formulae Sa/(reaction time × Sv), where Sa is the amount (nmole) of DCIP (2,6-Dichlorophenolindophenol) generated in a sample well between T (initial: 3 min) and T (final: 30 min) which was calculated from the standard curve, reaction time is the time difference (min) between T(initial) and T(final), and Sv is the sample volume (μl) added to each well. The SDH activity was reported as nmole/min/mg ER/mitochondrial protein.

### Statistical analysis

Statistical analysis was performed using Prism version 7.00 (GraphPad Software). Comparisons between two groups were performed using Student’s t-test (unpaired). When there were 3 or more groups, data were analyzed by one-way ANOVA (randomized) [35, 36] followed by Dunnett's post hoc multiple comparison test to determine differences between specific experimental groups and the control group [35]. Differences were considered significant with *P* < 0.05 and throughout the text, if a *P* value is ≤ 0.05, ≤ 0.01, ≤ 0.001, or ≤ 0.0001, it was flagged and represented with one, two, three, or four asterisks, respectively.

## Results

### Constitutive expression of CAMKK2 differed between HEK293 and HepG2 cells

**Fig. 1:**
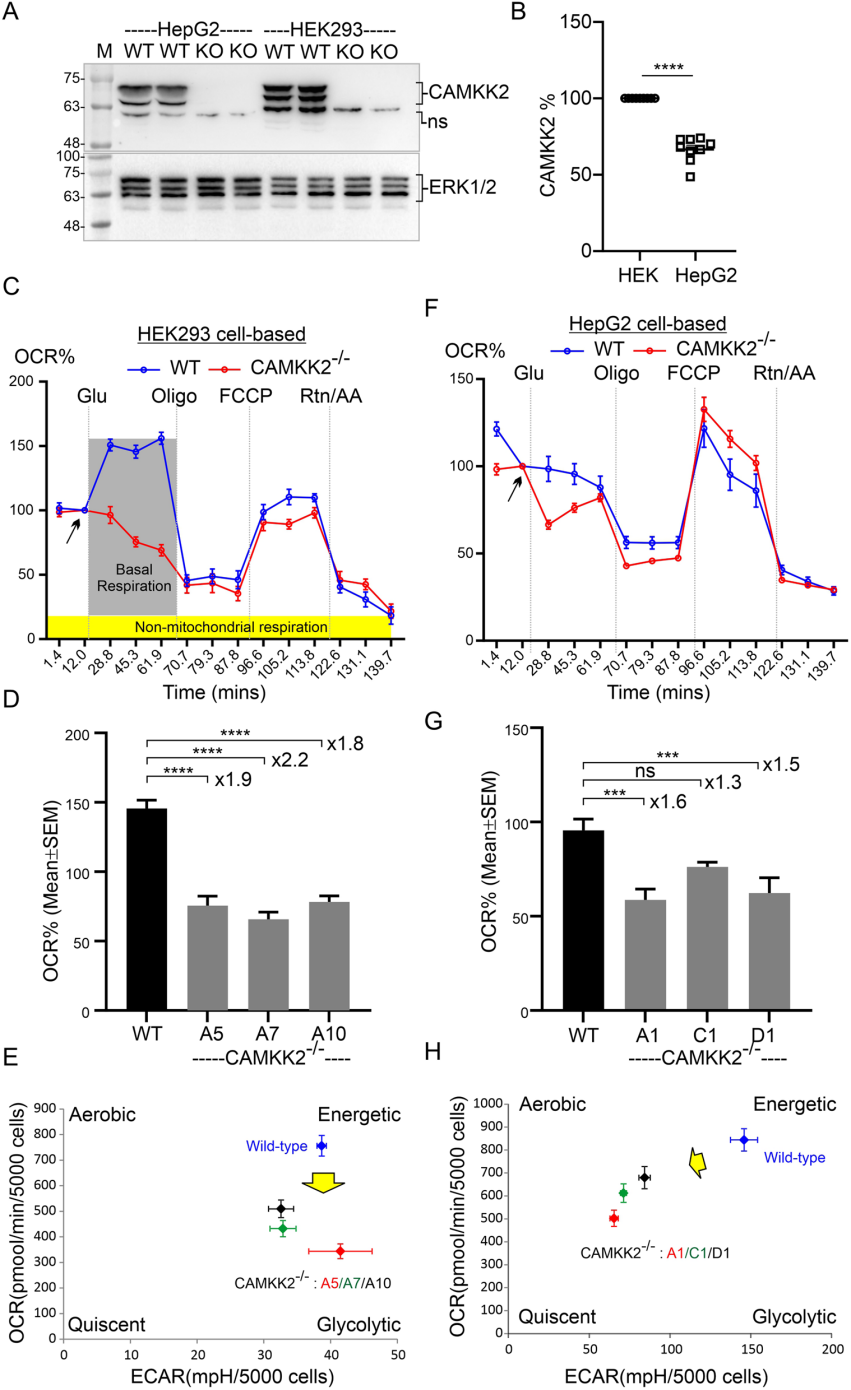
Cellular respiration in CAMKK2 deleted HEK293, HepG2 and EA.hy926 cell clones measured with an XF-24 extracellular flux analyzer. **A**: Immunoblots showing expression of CAMKK2 in HEK293 and HepG2 cells. WT: wild-type (parental), KO: CAMKK2 knockout (CAMKK2^−/−^), M: molecular weight ladder, and ns: nonspecific band. **B** Scatter plot showing CAMKK2 protein levels in HEK293 (HEK), HepG2 cells. The relative expression was normalized based on HEK293. Data presented as Mean ± SEM. N = 3 replicates from 3 independent experiments. Statistical significance from one-way ANOVA followed by multiple comparisons test. **C**, **F** Line graphs showing OCR kinetics at different time points following glucose/drug injections in the parental and CAMKK2 deleted HEK293 (**C**), and HepG2 (**F**) cell clones. Glu: glucose, Oligo: Oligomycin, FCCP: Carbonyl cyanide 4-(trifluoromethoxy), and Rtn/AA: rotenone/antimycin. The grey and yellow highlighted areas in D indicate basal respiration following injection of glucose and non-mitochondrial respiration following Rtn/AA injection, respectively. Data presented as Mean ± SEM, N = 10 replicates. Arrows indicate pre-glucose injection OCR rate set at 100 for normalization. This allows comparison between different biological replicates. **D**, **G** Bar graphs showing OCR (basal respiration) at 30 min after glucose injection. The basal respiration was calculated by subtracting non-mitochondrial respiration rate from 2nd rate measurement after glucose injection. Data presented as Mean ± SEM, N = 20 replicates from 2 independent experiments. Statistical significance from one-way ANOVA followed by multiple comparisons test. “**×**” indicates fold change. **E**, **H**: ECAR versus OCR plots after 30 min of glucose injection. Data presented as Mean ± SEM, N = 20 replicates from 2 independent experiments. The yellow arrows indicate a shift in the metabolic phenotype under CAMKK2 deleted condition

Previously, alternatively spliced (exon 14) CAMKK2 isoforms were detected as two distinct protein bands in the range of p70-75 kDa [17]. In this study, immunoblotting confirmed the presence of p70-75 CAMKK2 isoforms in parental HEK293 and HepG2 cells (Fig. 1A). Immunoblotting also confirmed complete loss of CAMKK2 expression in CAMKK2 deleted (CAMKK2^−/−^) HEK293 and HepG2 cell clones (Fig. 1A) as reported previously [16, 17]. Relative quantification revealed a significantly lower level of CAMKK2 proteins in HepG2 (66%) cells compared to HEK293 (100%) cells under basal conditions (Fig. 1B).

### CAMKK2 deficiency dampened cellular respiration in HEK293 and HepG2 cells

Cellular glucose metabolism was interrogated by simultaneous measurement of OCR and extracellular acidification rate (ECAR) using a Seahorse flux analyzer (mitochondrial function test). ECAR approximates glycolysis and OCR is an important metric for mitochondrial function [37]. The OCR vs ECAR plot provides a system-level snapshot of cellular metabolic function when mitochondria are engaged in oxygen consumption and energy generation through complex-V (ATP synthase) activity [37]. The mitochondrial function test revealed a significant decrease in the OCR/respiration within 30 min of glucose treatment to the glucose-starved CAMKK2^−/−^ HEK293 and HepG2 cell clones compared to parental cells (Fig. 1C–G, respectively). The ECAR vs OCR plot identified different cellular metabolic phenotypes under CAMKK2 deleted conditions that reflected a shift in the utilization of energy pathways to a more aerobic glycolytic/quiescent state within 30 min of glucose treatment (Fig. 1EH, yellow arrows). Interestingly, CAMKK2 deletion decreased ECAR in both cell types but the overall degree or extent of the decrease in ECAR exhibited some variability within the cell types which is possibly due to the metabolic differences which were highlighted in a study showing a difference in the glycolytic capacity (the difference between ATP in the presence and absence of 2DG normalized to vehicle) following treatment of HEK293 and HepG2 cells with 1 μM ellagic acid [38]. Overall, these results indicated dampened cellular respiration under CAMKK2 deletion conditions in both cell types, suggesting a universal metabolic effect (Table 2).

**Table 2:** Summary of the cell-type-specific differences involving bioenergetics, relative expression of SDHs, SDHs-multiprotein assembly, and PTMs of SDHs observed under CAMKK2 deletion conditions in HEK293 and HepG2 cells

Cell line	HEK293	HepG2
*Effect of CAMKK2 deletion*		
Tissue source	Embryonic kidney	Liver
Origin/disease	Transformed cell line	Hepatocellular carcinoma
Morphology	Epithelial	Epithelial-like
Basal cellular respiration	Decreased	Decreased
CI-IV respiration	Increased	Decreased
CII-IV respiration	Increased	Decreased
SDHA mRNA level	Decreased	Unaltered
SDHB mRNA level	Increased	Decreased
SDHC mRNA level	Unaltered	Unaltered
SDHA protein level	Increased	Unaltered
SDHB protein level	Increased	Decreased
SDHC protein level	Increased	Unaltered
SDHB-associated MPC	Shifted to the higher mol. weight	Shifted to the lower mol. weight
SDHB PTM state	Charged fractions altered	Charged fractions unaltered
SDH enzymatic activity	Increased	Decreased

### ER and mitochondrial organization differed between HEK293 and HepG2 cells

The ER/mitochondrial distribution in HEK293 and HepG2 cells was examined by TEM to assess cell-type-specific differences in mitochondrial distribution as well as to validate a subcellular enrichment process which was subsequently used to analyze isolated mitochondrial function. TEM images revealed a comparatively random distribution of ER and mitochondria in HEK293 cells compared to HepG2 cells (Additional file 2: Fig. S2A–B). In HepG2 cells, mitochondria were frequently sandwiched (yellow arrow) or partially encircled (cyan arrow) by rough endoplasmic reticulum structures (RER) with relatively increased contacts (red arrows) between these structures known as mitochondrial-associated ER membranes (MAMs) (Additional file 2: Fig. S2B). In contrast, MAMs were rarely observed in HEK293 cells. TEM-based images revealed the presence of both ER and mitochondrial structures in the enriched fractions (Additional file 3: Fig. S3A–D). The frequency of MAMs in the isolated ER/mitochondrial fraction was comparatively higher in HepG2 compared to HEK293 cells as previously observed in cells grown on nitrocellulose membrane (Additional file 3: Fig. S3D).

### Isolated mitochondrial function, specifically SDH (C-II) driven respiration, differed between CAMKK2 deleted HEK293 and HepG2 cells

**Fig. 2:**
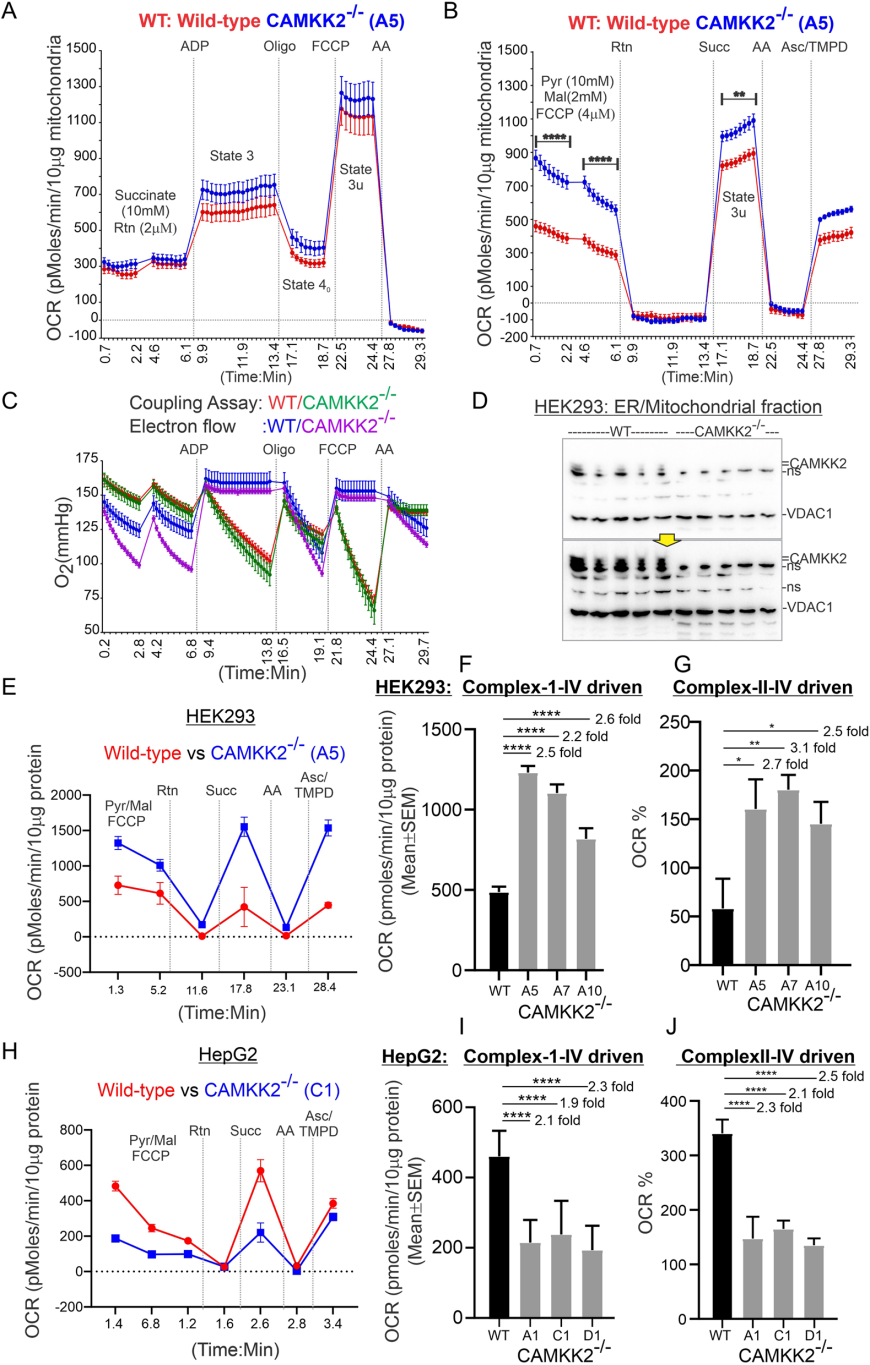
Coupling and electron flow assays to study OXPHOS functioning in enriched mitochondria from parental and CAMKK2 deleted HEK293 and HepG2 cells. (A-B): Line graphs showing OCR kinetics in coupling (**A**) and electron flow (**B**) assays performed simultaneously using 10 µg protein equivalent of enriched mitochondria in a Seahorse 24X flux analyzer. Final concentration of the inhibitors and substrates are mentioned in the text. The data were generated using a “point-to-point” mode in the Seahorse XF24 software package. The point-to-point displays OCR as a series of rates across the measurement period and can show changes of the rate across the measurement period [30]. The electron flow assay data presented in B is also provided as a “middle point” mode in (**E**, **H**) which is a preferred method for statistical comparison between groups (WT and CAMKK2^−/−^). The middle point mode shows a single OCR value for the measurement period which is the average of the point-to-point rates. Note that when the point-to-point rates are stable (relatively constant) across the measurement period, both point-to-point and middle point modes will provide an equivalent rate [30]. **C** Line graphs showing the O_2_ tension kinetics in the transient microchamber for 10 µg protein samples. **D** Immunoblots showing the relative abundance of CAMKK2 and VDAC1 in 10 μg of protein loaded in the Western blot to evaluate presence of equal amounts of proteins in the Seahorse assay. The yellow arrow indicates longer exposure of the top panel immunoblot. ns: nonspecific. **E**, **H** Line graphs showing OCR kinetics in the electron flow assay performed using 10 µg protein equivalent of enriched ER/mitochondrial fractions in a Seahorse 24X flux analyzer. Final concentration of the inhibitors and substrates are mentioned in the text. **F**, **I** Bar graphs showing uncoupled CI-IV driven OCR. N = 20 replicates from 2 independent experiments. **G**, **J** Bar graphs showing CII-IV mediated uncoupled OCR. Data were normalized by setting the OCR as 100% before rotenone injection. Statistical significance in F-G and I-J is from one-way ANOVA followed by multiple comparisons

We performed coupling and electron flow assays to assess mitochondrial function in isolated and enriched mitochondria derived from CAMKK2^−/−^ HEK293 and HepG2 cells (Additional file 3: Fig. S3) [17, 27]. The experiments described as the coupling assay examine the degree of coupling between ETS and OXPHOS, and this can distinguish mitochondrial function/dysfunction. In the coupling assay, the level of respiratory coupling was assessed by sequentially measuring OCR in the presence of succinate as a substrate and rotenone as a respiratory complex-I (CI) inhibitor (Fig. 2A, Additional file 1: Fig. S1). ADP, oligomycin, FCCP, and antimycin A were sequentially injected, and OCR measurements were taken after each injection (Fig. 2A). Addition of ADP increased OCR as expected (Fig. 2A). The ADP-activated state (Substrates + ADP + inorganic phosphate) at maximum oxygen flux is a measure of the capacity for oxidative phosphorylation (state-3) [39]. Subsequent injection of oligomycin inhibited ATP synthase (complex-V: CV) and decreased OCR (State 4_0_), however, addition of the uncoupler FCCP [40] increased OCR (State 3u) (Fig. 2A). In an uncoupled state at optimal uncoupler concentration, the maximum oxygen flux is an apparent measure of ETS excess capacity (state-3u) (Fig. 2A) [39]. The biological significance of ETS excess capacity (uncoupled respiration) over ADP-stimulated OXPHOS capacity cannot be explained at present but significant differences have been observed between human and mouse skeletal muscle mitochondria [39]. Finally, addition of the complex-III (CIII) inhibitor antimycin A decreased OCR (Fig. 2A). The absolute O_2_ tension (in mmHg) in the microchamber (microplate) was in the range of 75–175 mmHg (Fig. 2C) which is within the permitted range [30]. The coupling efficiency of oxidative phosphorylation (the percentage of respiration rate at a given mitochondrial membrane potential that is used for ATP synthesis) was measured by calculating the respiratory control ratio (RCR: State 3/State 4o) [41]. The coupling assay revealed no difference in the coupling efficiency (WT vs CAMKK2^−/−^ RCR; Mean ± SEM = 6.26 ± 0.12 vs 6.49 ± 0.14; *P* value 0.23 by unpaired t-test; N = 10 replicates from 2 independent experiments) of isolated enriched mitochondria derived from CAMKK2^−/−^ and parental cells.

The electron flow assay was designed to follow and interrogate each complex of the ETS (Fig. 2B, Additional file 1: Fig. S1). As oxidation of pyruvate/malate is mediated via CI, injection of rotenone inhibited this process, and respiration was halted. Injection of succinate allowed the mitochondria to respire via complex-II (CII), and OCR values increased. Electron flow was then inhibited at CIII by antimycin A, and respiration stopped as expected. Finally, addition of ascorbate and *N*,*N*,*N*′,*N*′-Tetramethyl-p-phenylenediamine (TMPD), which act as electron donors to CIV, elicited an increase in the OCR. In both assays (Fig. 2AB), equivalent protein (10 µg) loading in each well was confirmed by immunoblotting after completion of the Seahorse run using the protein lysates derived from the individual wells (Fig. 2D). Simultaneous immunoblotting using anti-CAMKK2 and VDAC1 revealed the absence of CAMKK2 in CAMKK^−/−^ HEK293-derived enriched mitochondrial fractions as expected and the relative amount of VDAC1 was similar (*p* > 0.05) between CAMKK^−/−^ and parental experimental sets indicating equivalent mitochondrial protein loading (Fig. 2D). In the electron flow assay, CII-IV mediated respiration was measured by setting the basal OCR value as 100% at the time point before rotenone injection. (Fig. 2E, H).

Electron flow analysis using 10 µg protein of enriched ER/mitochondrial fractions revealed a significant increase in the uncoupled state-3u [42] respiration in the presence of 10 mM pyruvate and 2 mM malate as substrates in CAMKK2^−/−^ HEK293 cell clones compared to parental cells (Fig. 2E–G). Both CI-IV and CII-IV-mediated respiration were significantly higher in the uncoupled state in isolated CAMKK2^−/−^ HEK293-derived enriched mitochondria compared to parental cells (Fig. 2FG). In contrast, state-3u respiration was significantly decreased in HepG2 cell clones compared to parental cells (Fig. 2H–J). Both CI-IV and CII-IV-mediated respiration was significantly decreased in CAMKK2^−/−^ HepG2 cell clones-derived mitochondrial fractions compared to parental cells (Fig. 2IJ). This indicates a cell-type-specific effect of CAMKK2 on isolated mitochondrial function and a yet uncharacterized factor may regulate the dissipation of promotive force in the FCCP-induced uncoupled state in a cell-type-specific manner. The biological relevance of respiration under the FCCP-induced uncoupled state is not clearly known, therefore, further elucidation of this difference is subject to future studies. Overall, these experiments revealed the effectiveness of the electron flow assay to perform functional analysis of the individual respiratory complexes, specifically CII-mediated respiration, in different cell-types which was further validated by the measurement of SDH enzymatic activity presented in Fig. 8. In summary, these results indicate that CAMKK2 loss differentially affected isolated mitochondrial function in a cell-type-specific manner with CII-mediated respiration increased in HEK293, but decreased in HepG2 cells (Table 2).

### CAMKK2 was detected in the enriched ER/mitochondrial fractions in both HEK293 and HepG2 cells

**Fig. 3:**
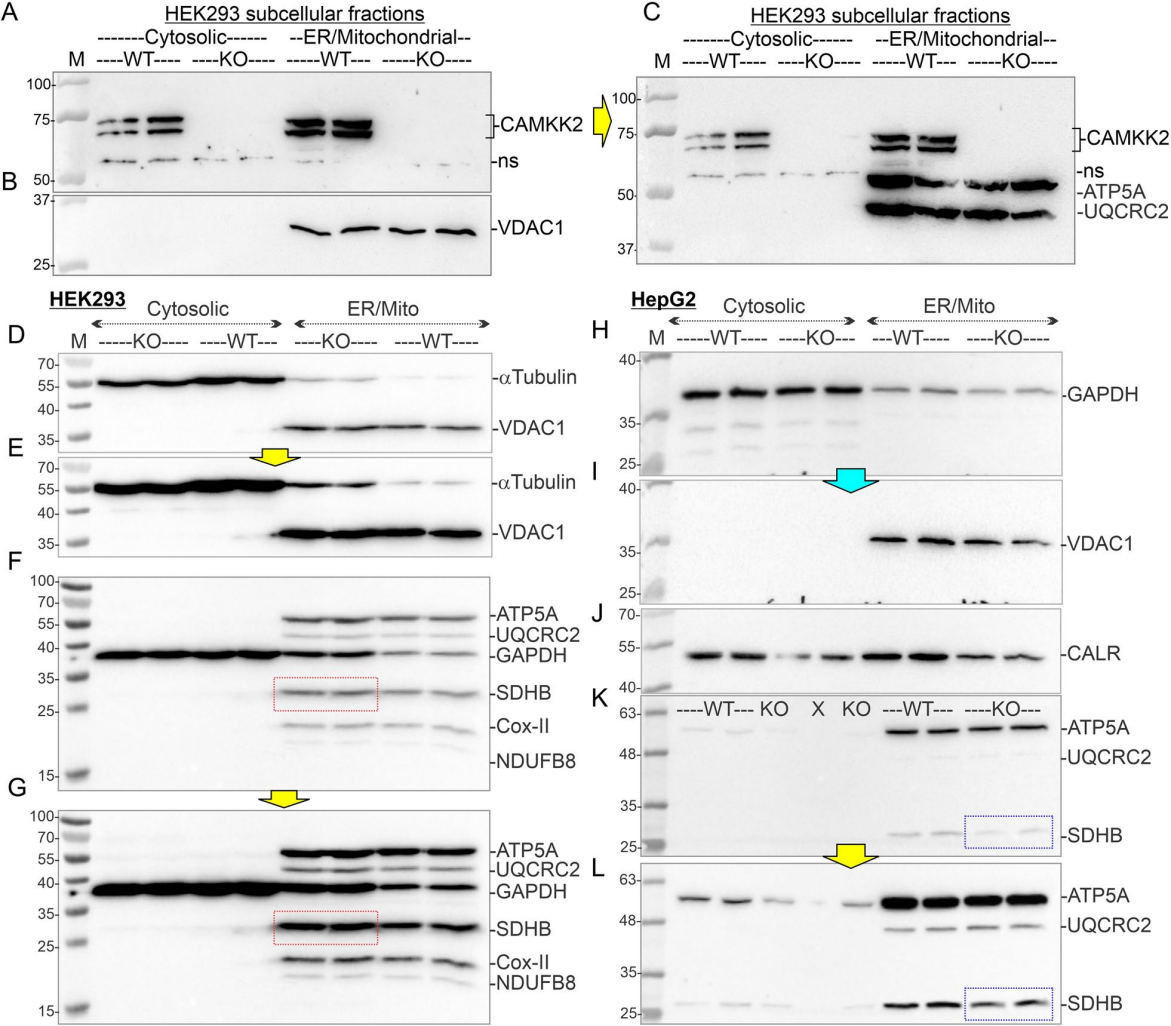
Fractionation of enriched ER/mitochondria. **A**–**C**: Immunoblots showing presence of CAMKK2, VDAC1, ATP5A and UQCRC2 proteins in subcellular fractions derived from HEK293 and HepG2 cells. Individual lanes under each category represent multiple replicates from a single set of experiments. WT: wild-type (parental), KO: CAMKK2 knockout, M: molecular weight ladder, and ns: nonspecific band. Yellow arrow indicates that the blot was incubated with a different set of antibodies without stripping. D–L Immunoblots showing abundance of cytosolic GAPDH and α-tubulin, mitochondrial ATP5A, SDHB, UQCRC2 and VDAC1, and ER-associated CALR [108] proteins in the subcellular fractions derived from parental (WT) and CAMKK2^−/−^ (KO) HEK293 (D–G) and HepG2 (H–L) cells, respectively. “ × ”: Blank lane. The yellow arrows indicate longer exposure of the corresponding immunoblots. The cyan arrow indicates incubation of the corresponding blot with anti-VDAC1 antibody after striping anti-GAPDH. The red and blue rectangles indicate relatively increased or decreased SDHB levels

The enriched ER/mitochondrial fractions [17, 27] were examined for the presence of CAMKK2 and were validated using mitochondria/cytosol-localized proteins as subcellular markers (Fig. 3A–C). Immunoblotting revealed the presence of CAMKK2 in the cytosolic and ER/mitochondrial fractions derived from HEK293 cells (Fig. 3A). The enrichment of mitochondria in the ER/mitochondrial fractions was confirmed by the presence of the mitochondrial solute carrier protein VDAC1, CV subunit ATP5A, and CIII subunit UQCRC2 (Fig. 3B, C, DE, I). The VDAC1 and OXPHOS-associated proteins, ATP5A and UQCRC2, were used as mitochondrial markers. Subsequent immunoblotting revealed the presence of GAPDH and α-tubulin in the cytosolic fractions in respective cell types (Fig. 3D–G, H. The anti-OXPHOS antibody cocktail used in this study (Table 1) consists of a mixture of antibodies specific to CI (NADH-Ubiquinone Oxidoreductase Subunit B8: NDUFB8), CII (Succinate Dehydrogenase Complex Iron-Sulfur Subunit B: SDHB), CIII (Ubiquinol-Cytochrome C Reductase Core Protein 2: UQCRC2), CIV (Mitochondrially Encoded Cytochrome C Oxidase II: MT-CO2), and CV (ATP synthase subunit alpha : ATP5A). The co-abundance of VDAC1 and OXPHOS proteins in the enriched ER/mitochondrial fraction indicated enrichment of mitochondria (Fig. 3D–G, H–L). The presence of ER structures was verified by using ER-specific CALR which was detected in both cytosolic and ER/mitochondrial fractions in HepG2 cells (Fig. 3J). Overall, these results indicated the effectiveness of the enrichment process. One striking observation was the comparative increase in CII-associated SDHB levels in CAMKK2^−/−^ HEK293-derived ER/mitochondrial fractions compared to parental cells (Fig. 3FG, red rectangles). In contrast, the SDHB level was relatively reduced in CAMKK2^−/−^ HepG2 cells compared to the parental cells (Fig. 3KL, green rectangles). The relative difference in the SDHB level correlated with the increased/decreased CII-driven respiration in the respective cell types (Fig. 2G, J).

### CAMKK2 deletion differentially altered OXPHOS and SDHB protein levels in HEK293 and HepG2 cells

**Fig. 4:**
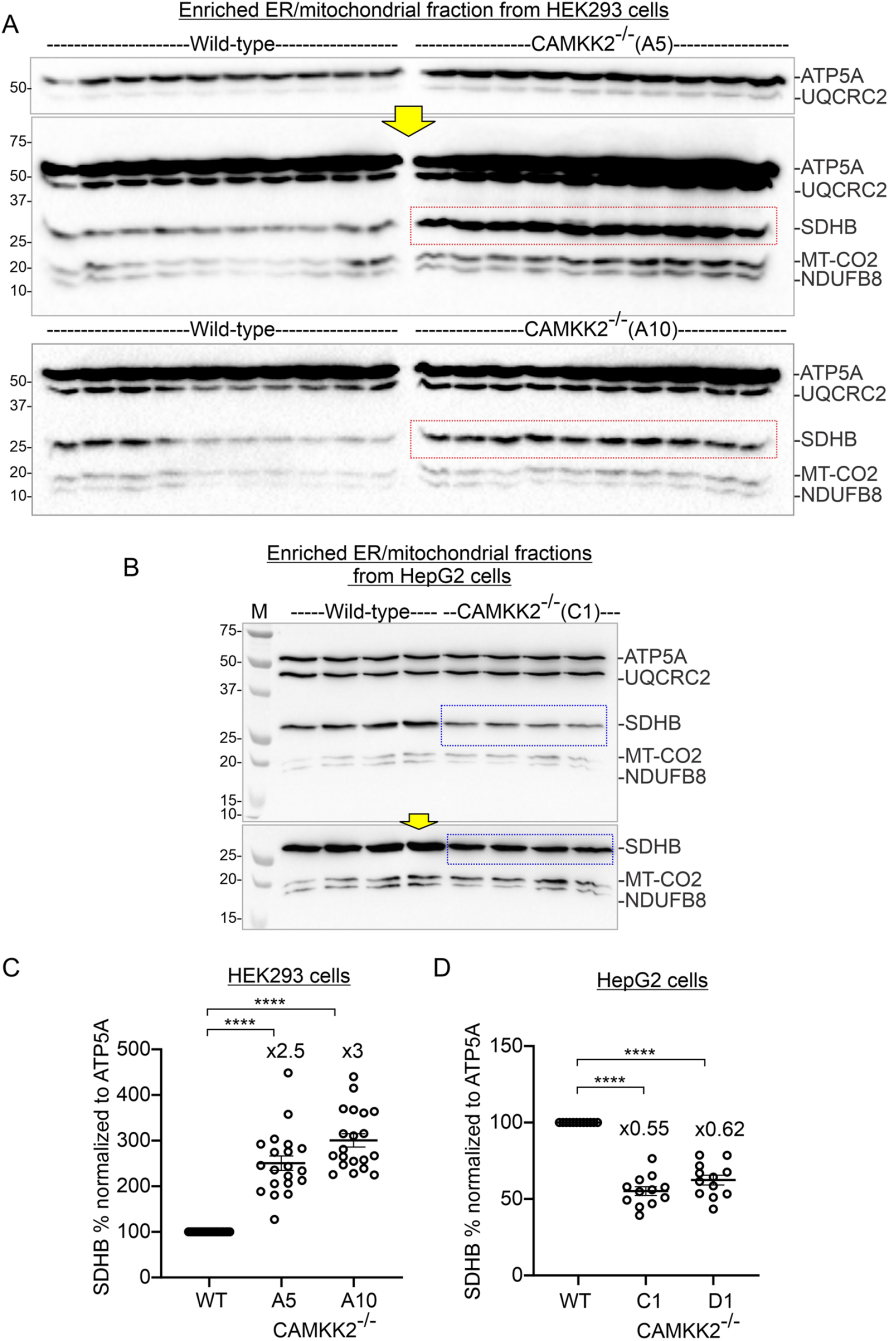
Effect of CAMKK2-deficiency on the abundance of OXPHOS-associated proteins in HEK293, EA.hy926, and HepG2 cell-derived enriched ER/mitochondrial fractions. **A**, **B** Immunoblots showing abundance of OXPHOS-associated proteins. The red rectangle marked areas indicate a relatively high level of SDHB in CAMKK2^−/−^ HEK293 cell clones (clone-A5 and -A10) compared to the parental cells. The blue rectangle marked area in **B** indicates a relatively low level of SDHB in CAMKK2^−/−^ HepG2 cell clone (clone-C1) compared to the parental cells. Yellow arrows indicate longer exposure of some segment of the top immunoblot to highlight bands that are underexposed. **C**, **D** Scatter plots showing relative level of SDHB in different CAMKK2 deleted cell clones. Statistical significance by one-way ANOVA followed by multiple comparisons (EG). “**×**” indicates fold change

The cell-type-specific difference in isolated mitochondrial function, specifically CII driven respiration, and the difference in the relative amount of SDHB in the ER/mitochondrial fractions under CAMKK2 deleted conditions (Figs. 2, 3) encouraged us to examine the effect of CAMKK2-deficiency on OXPHOS levels in both cell types using multiple cell clones (Fig. 4). Immunoblotting-based quantification revealed a significant and consistent increase in SDHB levels in the enriched ER/mitochondrial fractions derived from multiple independently selected CAMKK2^−/−^ HEK293 cell clones compared to parental cells (Fig. 4A, C red rectangles). The relative amount of NDUFB8 (CI), UQCRC2 (CIII), MTCO2 (CIV), and ATP5A (CV) also exhibited significant clonal variations within CAMKK deleted cell clones compared to parental HEK293 cells, however, the general trend was not consistent, and therefore, relative expression of these proteins was not quantified in the cell types as the inconsistency may be due to clonal variations (Additional file 4: Fig. S4A–D). Interestingly, in contrast to HEK293 cells, SDHB levels were significantly and consistently decreased in the same fraction of multiple independently selected CAMKK2^−/−^ HepG2 cell clones compared to parental cells (Fig. 4BD, blue rectangle; Table 2).

### SDHs mRNA levels differentially increased or decreased in CAMKK2 deleted HEK293 and HepG2 cells

**Fig. 5:**
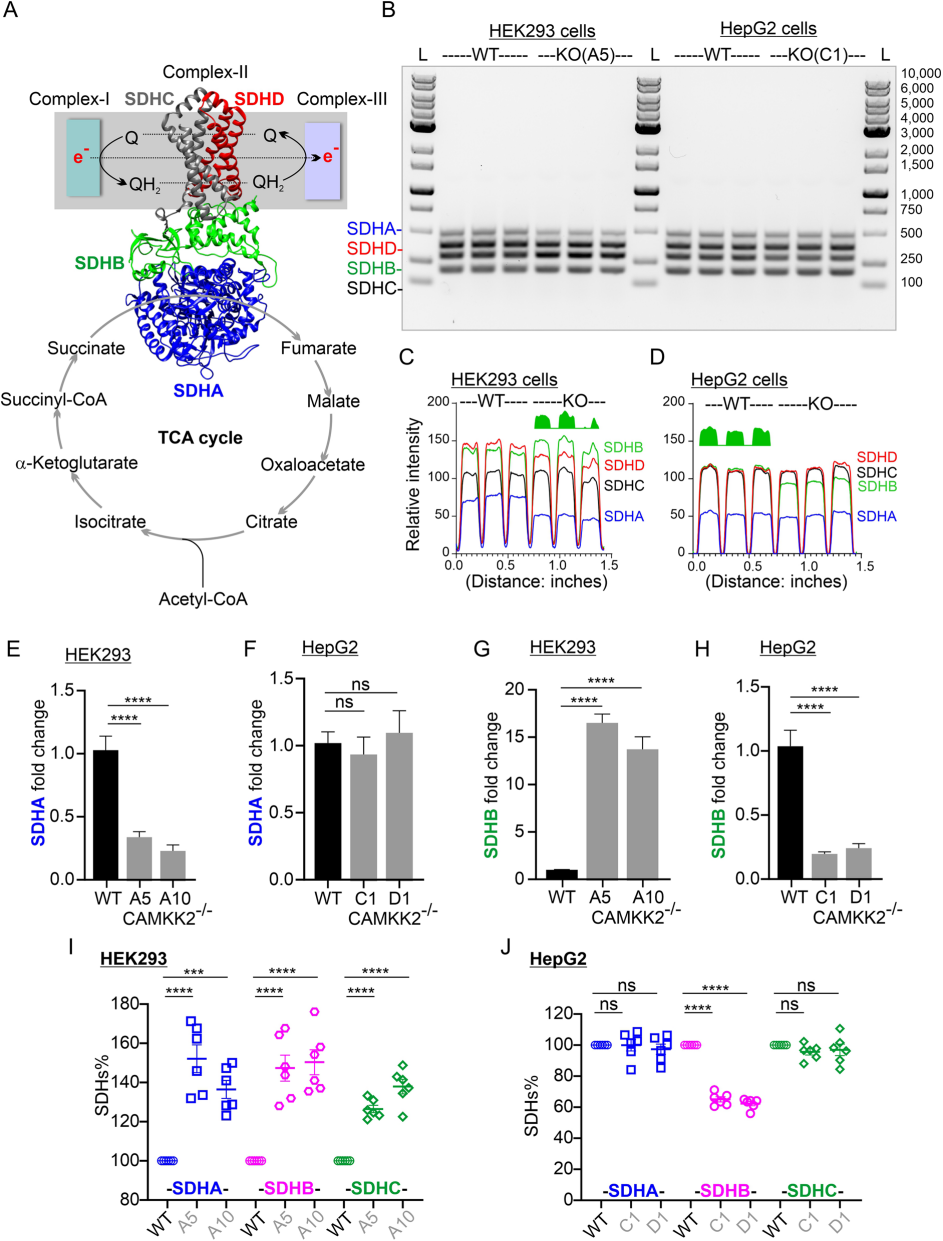
Cell-type-specific differential expression of SDHs mRNAs and proteins. **A** Diagram showing the involvement of the SDH protein complex (CII) in both the electron transport system and TCA cycle. The ribbon representation of the crystal structure of *Escherichia coli* SDH, analogous to the mammalian mitochondrial respiratory complex II, was based on Protein Data Bank (PDB) entry: 1NEK [109]. Molecular graphics were prepared using UCSF Chimera package [110]. **B** Agarose gel showing four subunits of SDH complex (SDHA, SDHB, SDHC, SDHD) and their specific PCR products simultaneously co-amplified by multiplex RT-PCR. WT: wild-type (parental), KO: CAMKK2 deleted HEK293 and HepG2 cells, L: molecular weight ladder. **C**, **D** The ImageJ-based plot profile of the SDH-specific bands presented in Figure B showing relative band intensities (arbitrary units) corresponding to respective gene expression level. The green space-filled areas indicate the relatively increased/decreased SDHB expression corresponding to an increased/decreased area under the curve in the respective cell types. **E**–**H** SDHA and SDHB fold change normalized to SDHC. Data presented as Mean ± SEM. N = 3 replicates from 2 independent experiments. Statistical significance from one-way ANOVA followed by multiple comparisons test. Ns: not significant (P > 0.05). **I**, **J** Scatter plots showing SDHA, SDHB and SDHC protein levels in parental (WT) and CAMKK2 deleted HEK293 (clone A5, 10) and HepG2 (clone C1, D1) cell clones. The SDHB was detected using mouse monoclonal anti-SDHB antibody obtained from Santa Cruz Biotechnology Inc. (Table 1)

CII is an enzyme complex bound to the inner mitochondrial membrane and composed of four subunits: the flavoprotein SDHA, iron-sulfur protein SDHB, and cytochrome b560 composed of SDHC and SDHD (Fig. 5A) [43, 44]. It is the only enzyme complex that participates in both the tricarboxylic acid (TCA) cycle and the electron transport chain (ETC) (Fig. 5A) [43, 44]. Therefore, we examined the mRNA levels of SDHs in HEK293 and HepG2 cells by multiplex RT-PCR. All four SDHs were co-amplified using four primer pairs that amplified four amplicons within a range of 202–508 nucleotides each separated by approximately 100 nucleotides (Table 1). Agarose gel electrophoresis and subsequent ImageJ-based plot intensity profiling of the PCR bands revealed cell-type-specific differential increases or decreases of SDHs (Fig. 5B–D). For example, the SDHB mRNA level was comparatively increased in HEK293 cells; in contrast, it was decreased in HepG2 cells (Fig. 5CD, green space filled areas under the curve). This correlated with an increased/decreased protein levels observed in these cell types, respectively (Figs. 3, 4). The SDHA mRNA level was considerably decreased in CAMKK2 deleted HEK293 cells compared to parental cells but remained relatively unaltered in HepG2 cells (Fig. 5CD). Further, the SDHC mRNA level remained relatively unaltered in both cell types whereas, SDHD showed some variation. The multiplex-RT-PCR-based observation encouraged us to quantify the relative expression of SDHB and SDHA mRNAs.

Based on the plot profile, we concluded the SDHC mRNA levels were relatively unaltered under different CAMKK2 deleted conditions. Therefore, to use it as a reference gene, we first examined the uniformity of SDHC mRNA expression under all conditions by designing a set of primers that amplified a larger 488 bp amplicon which was then gel purified and used to generate a standard curve using serial dilutions [31]. Absolute quantification using a nested primer set and a standard curve based on serial dilution of the copy numbers revealed no statistically significant difference between CAMKK2 deleted and parental HEK293 or HepG2 cells (Additional file 4: Fig. S4EF). This justified the use of SDHC as a reference gene to calculate the fold change of SDHB and SDHA. The SDHA level was significantly decreased in CAMKK2-deificient HEK293 cells compared to parental cells; in contrast, SDHA remained unaltered in HepG2 cells (Fig. 5EF). Interestingly, SDHB levels significantly increased in HEK293 cells but decreased in HepG2 cells under CAMKK2 deleted conditions (Fig. 5GH) and this correlated to a corresponding increase or decrease of the respective protein levels observed previously (Figs. 3, 4, Table 2).

### Subunits of SDH protein complex differentially increased or decreased in CAMKK2 deleted HEK293 and HepG2 cells

In Figs. 3–4, we demonstrated cell-type specific increases or decreases of SDHB using an anti-SDHB (Abcam) antibody that is part of a cocktail containing 5 monoclonal antibodies specific to different OXPHOS-associated proteins (Table 1). The Abcam anti-SDHB was generated using full length protein corresponding to Cow SDHB. To further validate this observation, we used another monoclonal anti-SDHB antibody that was raised against amino acids 1–280 representing full length SDHB of human origin (Table 1). Immunoblotting using a cocktail of anti-SDHA, anti-SDHB and anti-SDHC, all obtained from Santa Cruz Biotechnology, revealed a relative increase of all three proteins in CAMKK2^−/−^ HEK293 cells compared to parental cells (Additional file 5: Fig. S5AB, red dotted rectangles). Relative quantification revealed significant increases of SDHA, SDHB and SDHC proteins in CAMKK2^−/−^ HEK293 cell clones compared to parental cells (Fig. 5I). In contrast, SDHA and SDHC levels remained unaltered in CAMKK2^−/−^ HepG2 cell clones compared to parental cells, whereas the SDHB level was found significantly decreased (Fig. 5J, Additional file 6: Fig. S6A–D) as observed previously (Figs. 3KL, 4BD). Overall, these results indicate that mRNA levels of SDHs do not necessarily correlate with the translated protein levels in a cell-type-specific manner (Table 2).

### CAMKK2 deletion differentially altered mitochondrial OXPHOS and SDH-associated megacomplexes in HEK293 and HepG2 cells

**Fig. 6:**
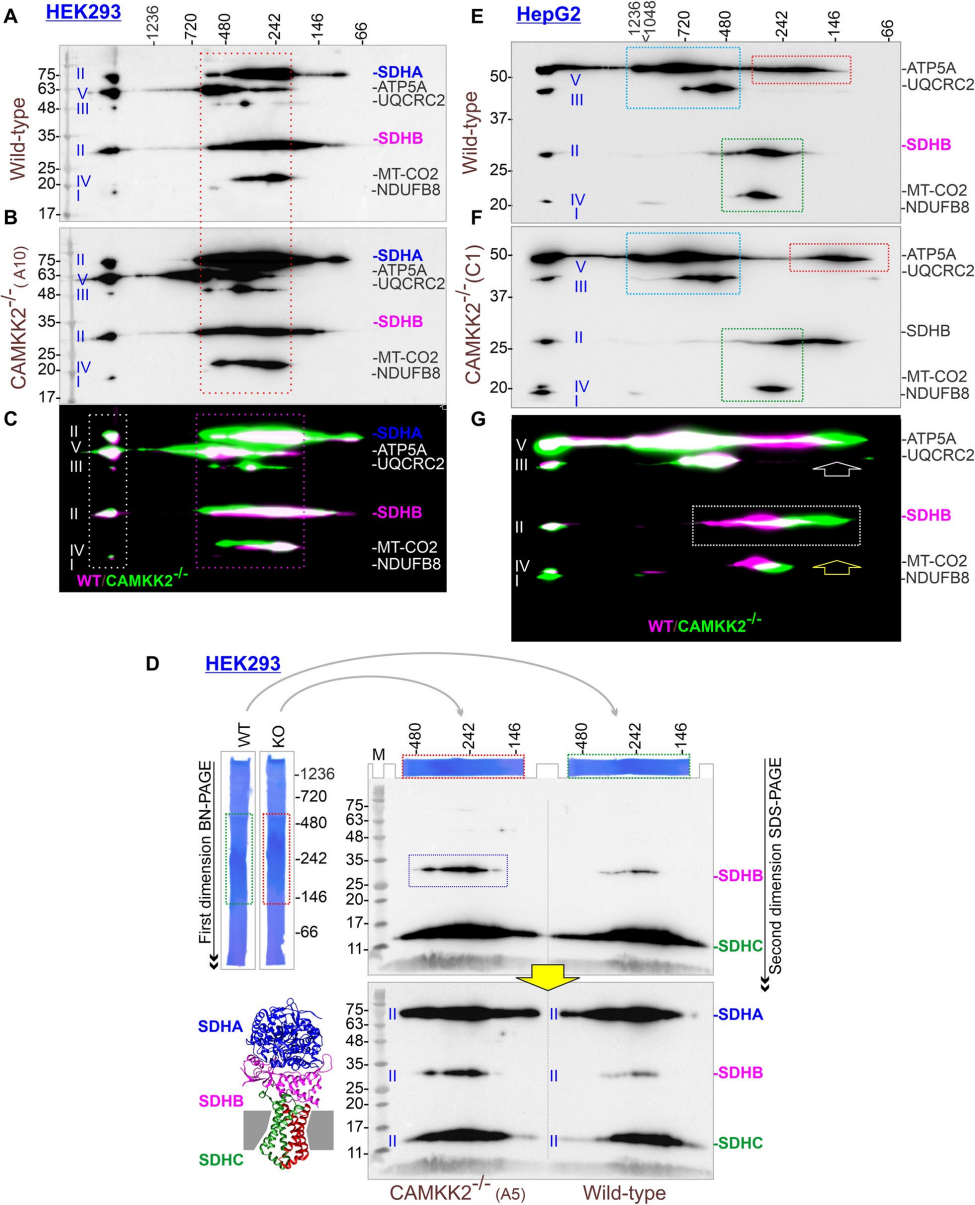
Cell-type-specific effect of CAMKK2 loss-of-function on the SDH and OXPHOS-associated MPCs. **A**, **B**, **E**, **F** Immunoblots showing OXPHOS-associated MPCs in the parental and CAMKK2^−/−^ HEK293 cell clone-derived mitochondria. The immunoblot was generated by simultaneous use of a cocktail of six antibodies (Table 1). **C**, **G** Immunoblots in AB and EF were false colored and overlaid to show relative amount\shift of OXPHOS-associated protein complexes. Vertical alignment of OXPHOS-associated proteins indicates association in multiprotein complexes that co-migrated during first-dimension native PAGE. **D** Immunoblots showing relative abundance of SDHA, SDHB and SDHC in complex II. The vertical alignment of SDHA, SDHB and SDHC indicates their association in a single megacomplex. Interpretation of colored markings and arrows: **A**–**C** Red/pink rectangles: potential megacomplex involving different OXPHOS complexes. White dotted rectangle: a > 1200 kDa MPCs potentially associated with different OXPHOS complexes to form respirasome structures. **E**–**G** Blue rectangles: differential oligomerization of ATP5A and UQCRC2. Green rectangles: differential shift of MT-CO2 MPCs. Red rectangle: differential shift of ATP5A. White rectangle: vertical alignment of SDHB MPCs. White and yellow arrow: relative abundance or differential shift of respective MPCs. **D** Grey arrows: placement of excised first dimensional BN-PAGE gel on the second dimension SDS-PAGE. Yellow arrow: Incubation of the same blot with a different antibody without stripping. Blue dotted rectangle: relative abundance of SDHB

Two-dimensional BN-PAGE/SDS-PAGE was used to study OXPHOS-associated multiprotein complexes (MPCs) [45–47]. The OXPHOS-associated MPCs exhibited a considerable difference between CAMKK2^−/−^ and parental HEK293 cell-derived mitochondria (Fig. 6A–C) due to the alterations in the relative abundance of OXPHOS-associated proteins as highlighted in Figs. 3and 4 and Additional file 5: Fig. S5AB. The BN-PAGE/SDS-PAGE is not quantitative for the relative abundance of MPCs between control (parental) and experimental (CAMKK2^−/−^) groups due to multiple variables but it may reflect an overall difference. One of the variables may be due to the fact that though the control and experimental proteins were resolved in the same first dimension native PAGE, proteins may be unintentionally lost during excision of the first dimension BN-PAGE gel strips. Another factor is that the immunoblots derived from the second-dimension SDS-PAGE are separately incubated with primary/secondary antibodies and detected individually using chemiluminescence under different sets of exposures to highlight under/over saturated bands within a broad dynamic range due to high vs low abundant proteins. However, if all conditions are kept nearly uniform, a reasonable comparison can be made by careful quantification of the immunoblots. False-colored overlaid images of 2D-BN-PAGE separated OXPHOS-associated protein complexes revealed an overall shift of OXPHOS-associated MPCs to a higher molecular weight region, and the abundance of SDHs in CAMKK2^−/−^ HEK293 mitochondria compared to parental cells (Fig. 6C). Dimeric and oligomeric ATP synthase are essential for maintaining mitochondrial ultrastructure and function [48]. Relatively increased abundance of oligomeric ATP5A in the range of ~ 480–1200 kDa in CAMKK2^−/−^ HEK293 mitochondria compared to the parental mitochondria may indicate increased association with other complexes or interactive protein and may thereby provide a functional advantage. The SDHB/SDHA (CII), ATP5A (CV), UQCRC2 (CIII), MT-CO2 (CIV)-associated MPCs were vertically aligned in the 242–720 kDa range (Fig. 6A–C, red and pink dotted rectangle). The vertical alignments of these complexes may represent the formation of a respiratory megacomplex structure which was relatively improved in CAMKK2^−/−^ HEK293 cells compared to parental cells due to increased abundance of OXPHOS-associated proteins (Additional file 6: Fig. S6AB). The vertical alignment of ATP5A (CV), MT-CO2 (CIV), UQCRC2 (CIII), and SDHB/SDHA (CII)-associated MPC in a very high molecular weight region (> 1200 kDa) may indicate a potential respirasome supercomplex (Fig. 6C, white dotted rectangle).

In order to reduce the variability in relative quantification of CII assembly during BN-PAGE/SDS-PAGE, we excised the first-dimension BN-PAGE gel at 146–480 kDa region containing MPCs from the parental and CAMKK2^−/−^ mitochondria and loaded in the second-dimension SDS-PAGE and subsequently transferred to a single nitrocellulose membrane and both samples were immunoblotted and visualized together (Fig. 6D, grey arrows). This strategy allowed us to remove few variables. Immunoblots revealed the perfect vertical alignment of SDHA, SDHB, and SDHC indicating their association in the same complex (Fig. 6D). The relative abundance of CII, specifically SDHB was more prominent in CAMKK2^−/−^ HEK293 mitochondria compared to the parental mitochondria (Fig. 6D, blue dotted rectangle). The ratio of SDHA versus SDHB versus SDHC in CAMKK2^−/−^ HEK293 mitochondria is 5.4 versus 1.2 versus 3.4 compared to 5.8 versus 0.5 versus 3.7 in parental mitochondria. This indicates that there were approximately 2 × more SDHB protein molecules associated with other SDHs in MPCs which may be responsible for the increased efficiency of CII-mediated reparation under CAMKK2 deletion condition.

CAMKK2 deficiency in HepG2 cells exhibited the opposite effect in the assembly of OXPHOS-associated MPCs compared to HEK293 cells. Overall, the ATP5A and UQCRC2-associated MPCs were aligned but shifted to relatively lower molecular weight regions in CAMKK2^−/−^ HepG2 mitochondria compared to parental cells (Fig. 6E–G, red rectangles, and white arrow). Also, the SDHB-associated MPC shifted to a relatively low molecular weight region in CAMKK2^−/−^ HepG2 mitochondria compared to the parental mitochondria (Fig. 6G, white rectangle), whereas the MT-CO2-associated MPCs also shifted to a lesser extent (Fig. 6E–G, pink and white rectangles). Thus, the exact vertical alignment of SDHB and MT-CO2-associated MPCs as previously observed in HEK293 cells was abolished in CAMKK2^−/−^ HepG2 mitochondria compared to parental mitochondria (Fig. 6C, H–J, pink rectangles). Further, to establish the cell-type-specific differential shift of SDHB-associated MPCs, we performed BN-PAGE analysis by loading both parental and CAMKK2 deleted HEK293 and HepG2 cell lysates simultaneously in the same first dimension native gel (Additional file 7: Fig. S7) and the immunoblotting was performed on second dimension SDS-PAGE-derived blots using anti-SDHA/SDHB antibodies obtained from Abcam and Santa Cruz Biotechnology, respectively (Table 1, Additional file 7: Fig. S7). Immunoblotting revealed a significantly increased abundance of SDHB-associated MPCs vertically aligned with SDHA-associated MPCs in CAMKK2^−/−^ HEK293 mitochondria compared to parental mitochondria (Additional file 7: Fig. S7A, B, H, green rectangles) and in addition, there was an overall shift of SDHA/SDHB-associated MPCs to a higher molecular weight region under CAMKK2 deleted condition (Additional file 7: Fig. S7EG, yellow arrows). In contrast, the SDHB was significantly less abundant in the SDHA associated MPCs in CAMKK2^−/−^ HepG2 mitochondria compared to parental mitochondria (Additional file 7: Fig. S7C-D, H, green rectangles) and SDHA/SDHB-associated MPCs shifted to a lower molecular weight region under CAMKK2 deleted condition (Additional file 7: Fig. S7F-G, red arrows). Overall, these data indicate improved CII MPCs in HEK293 cells compared to HepG2 cells which correlated with functional improvement under CAMKK2 deleted conditions.

### CAMKK2 loss altered PTMs (charged fractions) of OXPHOS, specifically SDHB, in a cell-type-specific manner

**Fig. 7:**
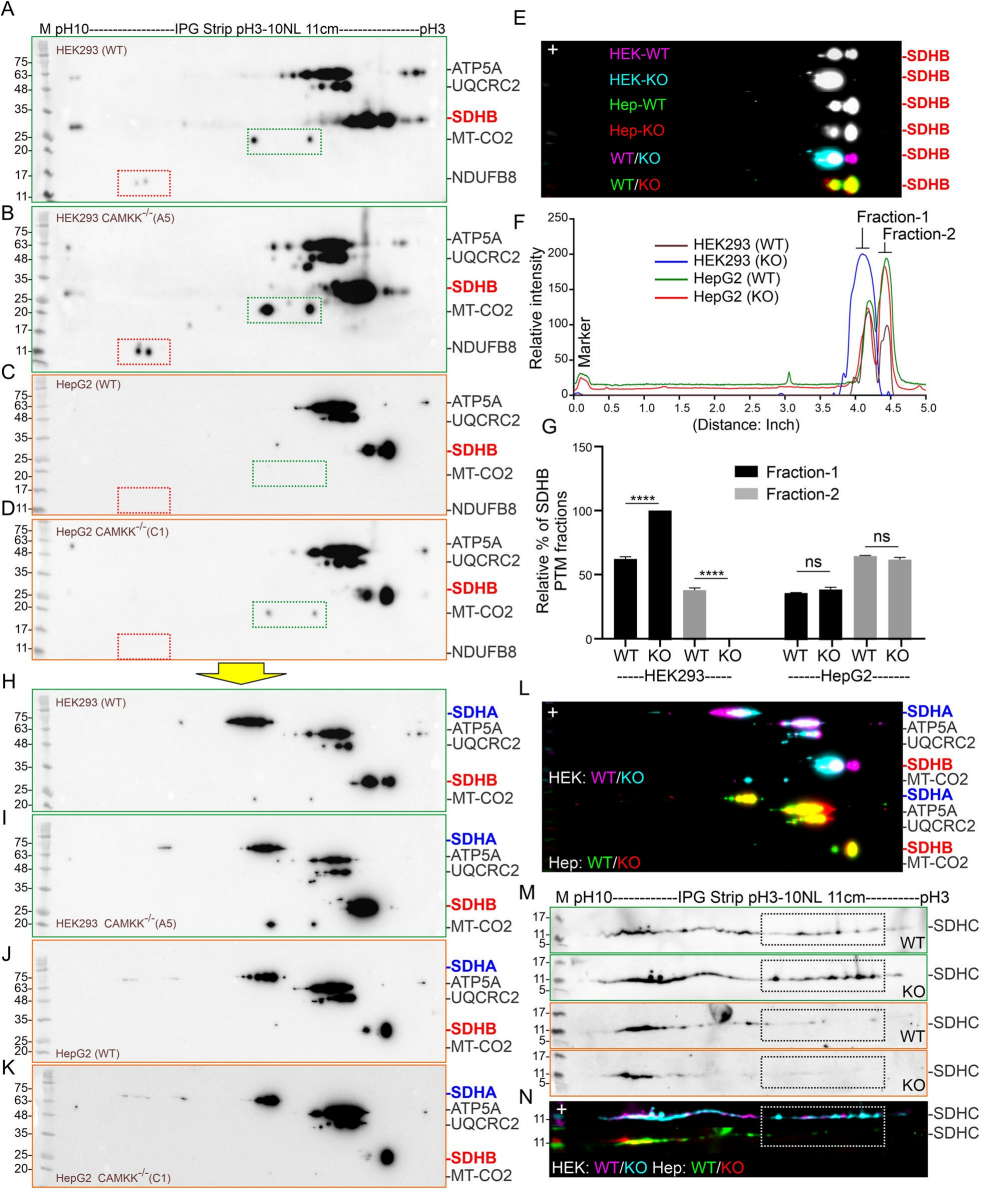
Loss of CAMKK2 differentially affected PTMs of OXPHOS, specifically SDHA in a cell-type-specific manner. **A**–**D** Immunoblots showing charged fractions of OXPHOS proteins. **H**–**K** The immunoblots presented in **A**–**D** were treated with a rabbit monoclonal anti-SDHA antibody. Therefore, the previously detected proteins using mouse monoclonal anti-OXPHOS antibodies may show some variation compared to A-D due to loss of mouse antibodies after prolonged incubation and washing. **E**, **L** The immunoblots presented in A-D (only SDHB fractions) and H–L were false colored and overlapped. “+”: pH/pI-10. **F** Plot profiles of the SDHB charged fractions. Fraction 1and 2 indicates same protein with different sets of modifications. **G** Bar graphs showing relative percentage of fraction 1 and 2 of SDHB protein. Statistical significance from one-way ANOVA followed by multiple comparisons. **M**–**N** Immunoblots showing charged fractions of SDHC. **N** False colored overlay of immunoblots presented in M. Interpretation of colored rectangles and arrows: Difference in charged fractions of MT-CO2 (green dotted rectangles), NDUFB8 (red dotted rectangles) and SDHC (black/white dotted rectangles)

We performed two-dimensional IFE/SDS-PAGE to study differential PTMs of OXPHOS and SDHs to understand the cell-type-specific difference in mitochondrial function. Immunoblotting followed by IEF/SDS-PAGE using anti-OXPHOS antibodies revealed a considerable difference in SDHB PTMs in CAMKK2^−/−^ HEK293 mitochondria compared to parental mitochondria (Fig. 7AB, E–G). The basal isoelectric point of SDHB (protein ID: ENSP00000364649.3) is 9.03, but IEF revealed the appearance of 2 major fractions at pI/pH 3–4 (designated as fraction-1 and 2) in both HEK293 and HepG2 cells (Fig. 7A-D, E–G, L). Relative quantification revealed a significant increase of fraction-1 and a corresponding decrease of fraction-2 SDHB in CAMKK2^−/−^ HEK293 mitochondria compared to parental mitochondria. In contrast, the relative amount of both fractions remained unaltered in HepG2 mitochondria under CAMKK2 deleted conditions. The pI of SDHA (protein ID: ENSP00000264932.6) and SDHC (protein ID: ENSP00000364649.3) are 6.39 and 6.13, respectively. Interestingly, both SDHA and SDHC exhibited cell-type-specific differences in the charged fraction under native as well as CAMKK2 deleted conditions; however, the difference was not as considerable as compared to SDHB (Fig. 7A-D, H–K, L, M–N). Further, the OXPHOS-associated proteins also exhibited considerable cell-type-specific differences, for example, the MT-CO2 and NDUFB8 (Fig. 7A–D, red and green rectangles). Overall, these datasets indicate cell-type-specific differential PTMs of OXPHOS, specifically SDHB protein modification under CAMKK2 deleted conditions which may account for the cell-type-specific mitochondrial functional difference (Table 2).

### Knockdown of SDHB in CAMKK2^−/−^ HEK293 cells significantly decreased SDH enzymatic activity

**Fig. 8:**
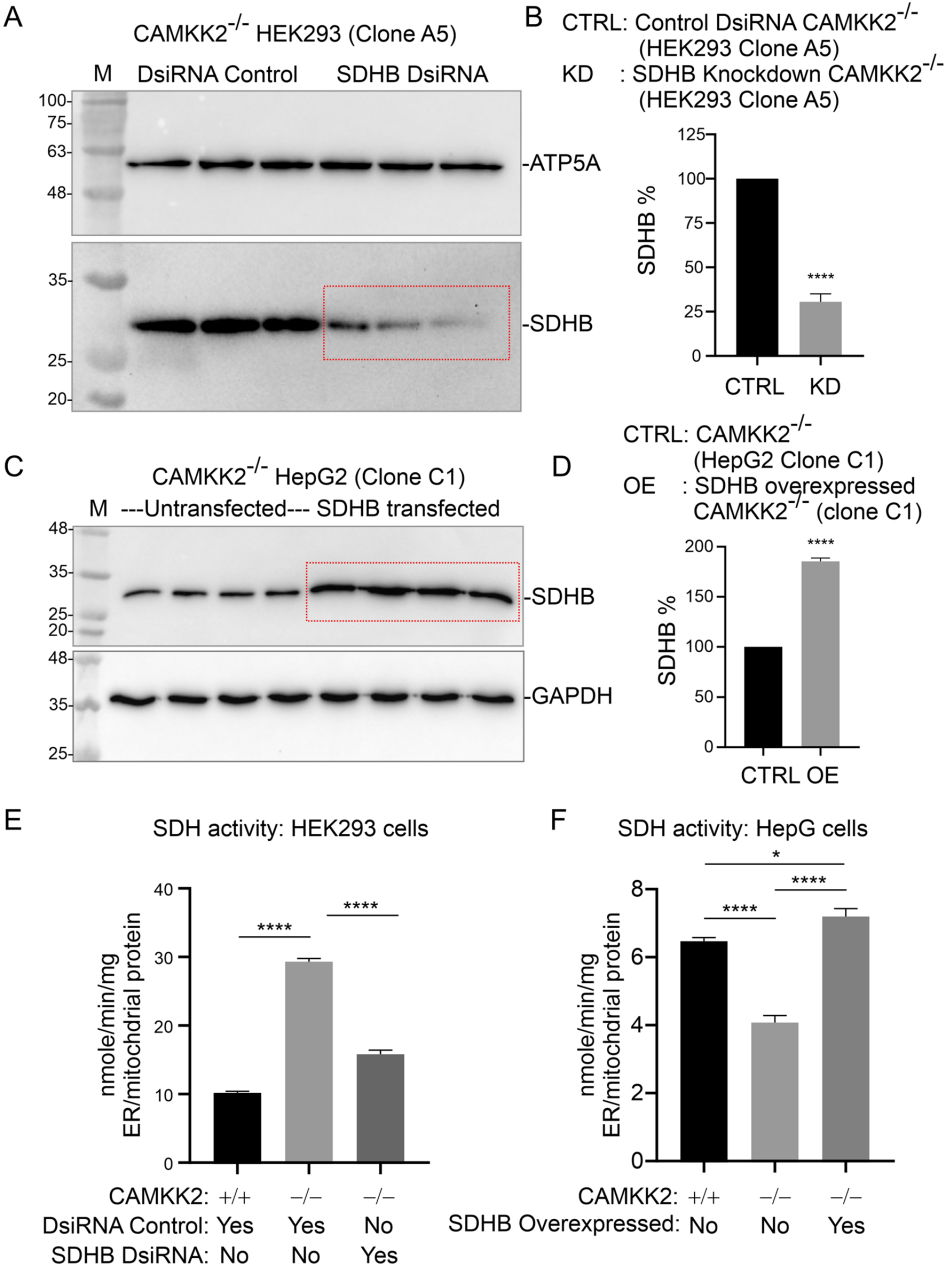
Knockdown and overexpression of SDHB in CAMKK2^−/−^ HEK293 and HepG2 cells, respectively. **A**, **C** Immunoblots showing ATP5A (**A**), GAPDH (**B**), and SDHB (**A**, **B**) protein levels in the SDHB knockdown (**A**) and SDHB overexpressed ER/mitochondrial fractions derived from CAMKK2^−/−^ HEK293 and HepG2 cells, respectively. **B**, **D** Bar graphs showing the relative amount of SDHB protein in the SDHB knockdown (**B**) and SDHB overexpressed ER/mitochondrial fractions derived from CAMKK2^−/−^ HEK293 and HepG2 cells, respectively. N = 6 replicates from 2 independent experiments. Data represented as Mean ± SEM. Statistical analysis by t-test (unpaired). **E**, **F** SDH enzymatic activity in the enriched ER/mitochondrial fractions derived from parental, CAMKK2^−/−^, and CAMKK2^−/−^ + SDHB knockdown/overexpressed HEK293 (**E**) and HepG2 (**F**) cells, respectively. N = 10 replicates from 2 independent experiments. Statistical significance from one-way ANOVA followed by multiple comparisons

In Figs. 2–5, we demonstrated that CAMKK2 deletion in HEK293 cells significantly increased SDHA, SDHB and SDHC protein levels which correlated to an increase in CII-mediated respiration compared to parental cells. Based on this, we hypothesized that knockdown of SDHB in CAMKK2^−/−^ HEK293 cells would reverse this phenotype. DsiRNA targeted to exon 3 was used to knockdown SDHB in CAMKK2^−/−^ HEK293 cells (Table 1). Immunoblotting revealed a significant reduction (~ 70%) of SDHB protein level in SDHB-targeted DsiRNA transfected CAMKK^−/−^ HEK293 cells compared to control DsiRNA transfected cells (Fig. 8AB). Enzymatic activity assay using enriched ER/mitochondrial fractions revealed a significant reduction in SDH activity in CAMKK2^−/−^/SDHB knockdown mitochondria compared to CAMKK2^−/−^ mitochondria (Fig. 8E). Also, the significantly increased SDH enzymatic activity observed in isolated CAMKK2^−/−^ mitochondria compared to parental HEK293 mitochondria (Fig. 8E) supported our previous flux analysis-based experiments that measured CII-mediated respiration (Fig. 2G). Overall, these findings indicate that a CAMKK2 loss-of-function-mediated increase in SDHB protein content is responsible for the enhanced mitochondrial function.

### Overexpression of SDHB in CAMKK2^−/−^ HepG2 cells significantly increased SDH enzymatic activity

In contrast to HEK293 cells, CAMKK2 deletion in HepG2 cells significantly decreased the SDHB protein level, which corresponded with the decrease in CII-mediated respiration compared to parental cells (Figs. 2–5). In order to see if an increase in SDHB protein level in CAMKK2^−/−^ HepG2 cells would reverse this phenotype, we overexpressed SDHB in CAMKK2^−/−^ HepG2 and measured SDH enzymatic activity in the enriched ER/mitochondrial fractions. Immunoblotting revealed a significant increase (~ 1.85 fold) in the SDHB protein level in a pool of SDHB overexpressed CAMKK2^−/−^ HepG2 cells compared to untransfected cells (Fig. 8CD). Enzymatic activity assay using enriched ER/mitochondrial fractions revealed a significant increase in SDH activity in CAMKK2^−/−^/SDHB overexpressed HepG2 mitochondria compared to CAMKK^−/−^ HepG2 mitochondria (Fig. 8F). Also, a significant decrease in SDH enzymatic activity was observed in isolated CAMKK2^−/−^ HepG2 mitochondria compared to parental mitochondria (Fig. 8F) supporting the previous flux analysis-based experiments which measured CII-mediated respiration (Fig. 2J). Overall, these findings indicate that the CAMKK2 loss-of-function-mediated decrease in SDHB protein content in HepG2 cells is responsible for the dampened mitochondrial function.

## Discussion

In this study, we demonstrated that inhibition of mitochondrial respiration during glucose metabolism is a universal phenotype under CAMKK2 deleted conditions irrespective of the organ or tissue-specific origin of the cell-types (Kidney: HEK293, Liver: HepG2). Interestingly, this universal suppression of respiration was not reciprocated in the isolated mitochondrial function which exhibited cell-type-specific differential respiratory kinetics under CAMKK2 deleted conditions compared to the kinetics of corresponding unperturbed parental cells. For example, the respiration of isolated mitochondria in an uncoupled state (state-3u) was increased in CAMKK2^−/−^ HEK293compared to parental cells, whereas, it was decreased in CAMKK2^−/−^ HepG2 cells compared to parental cells. Furthermore, we have shown that cell-type-specific increases or decreases in the mRNA as well as protein levels of nuclear-encoded SDHs (CII), specifically SDHA, SDHB, and SDHC, correlated with a corresponding increase or decrease of CII-mediated respiration in the respective cell-types. In addition, we demonstrated that the cell-type-specific effect on mitochondrial function under CAMKK2 deletion condition is associated with post-translational modification of OXPHOS proteins, including SDHs, specifically SDHB, and their assembly in supercomplex/megacomplex structures in the respective cell types. Overall, this study provided a unique mechanistic insight into CAMKK2-SDHs mediated regulation of mitochondrial bioenergetics which may provide the mechanistic basis for organ/tissue/cell-type-specific metabolic reprogramming.

CAMKK2 deletion affected both transcription and translation of nuclear-encoded SDHs in a cell-type-specific manner. The altered mRNA level may be due to increased/decreased transcriptional activity or mRNA stability. Similarly, the altered protein level may be due to increased/decreased translation or altered protein half-life due to PTMs. Interestingly, the cell-type-specific increase or decrease of SDHs mRNA levels was not reciprocated in a corresponding increase or decrease in the protein levels (Fig. 5), suggesting the regulatory mechanisms are operating at multiple levels. For example, under CAMKK2 deleted conditions, SDHC mRNA levels remained unaltered in both cell types but the SDHC protein level was considerably increased in HEK293 cells, whereas, in HepG2 cells, it remained unaltered. Also, CAMKK2 loss significantly reduced SDHA mRNA in HEK293 cells, in contrast, the protein level was considerably increased. On the other hand, both SDHA mRNA and protein levels remained unaltered in HepG2 cells. The reciprocal relation between mRNA, protein level and functional consequence (CII-mediated respiration) was only fulfilled in the case of SDHB, where an increase/decrease in SDHB mRNA level was reciprocated in a corresponding increase/decrease in the protein level as well as enhanced/dampened CII activity in the respective CAMKK2 deleted cell types. In addition, the cell-type-specific SDHB knockdown/overexpression-based study further validated this cause-and-effect relationship involving altered SDHB level leading to an altered CII activity under CAMKK2 loss-of-function conditions (Fig. 8). As SDHB is a mitochondrially localized protein, the relative increase or decrease in the SDHB protein level under CAMKK2 deficient condition was reflected in an increased/decreased abundance of the protein in the ER/mitochondrial fraction in the respective cell types. The exact mechanism of this cell-type-specific potential transcriptional/translational regulation is not known at this stage of the study but a hypothesis can be conferred based on the evidence in the existing literature. The differential transcriptional regulation can be mediated by transcription factors regulated by the cell-type-specific difference in CAMKK2 downstream signaling. Activated CAMKK2 directly phosphorylates multiple downstream effectors including CAMK1, CAMK4, AMPKα, and SIRT1 [2]. The transcriptome data available in the HPA database indicates considerable difference between HEK293 and HepG2 cells in the CAMKK2 downstream effector’s mRNA levels, and therefore, may support this hypothesis. For example, the normalized RNA expression values for CAMK1, CAMK4, AMPKα (PRKAA1) and SIRT1 is 21.1, 2.6, 7.9, and 10.9 in HEK293 cells whereas it is 7.4, 0, 8.8, and 8.2 in HepG2 cells, respectively. AMPKα phosphorylates and activates transcription factors PGC1α (peroxisome proliferator-activated receptor γ coactivator 1 α) [49] and NRF2 (respiratory factor 2) [50]. PGC1α is a master regulator of mitochondrial biogenesis [6] and regulates energy metabolism by modulating the expression of genes involved in oxidative phosphorylation via co-activation of NRF1/2 [51, 52]. Both NRF-1 and -2 are transcriptional regulators of SDHs [53]. Therefore, CAMKK2-AMPKα-PGC1α-NRF1/2 pathway may be a potential mediator of the transcriptional regulation of SDHs. Interestingly, the reduced level of PGC1α mRNA observed in Camkk2 knockout mouse hepatocytes supports this hypothesis [54]. On the other hand, constitutively expressed CAMK1 and CAMK4 may activate the nuclear transcription factor CREB [55]. An isoform of CAMKI (CAMK1δ) has been shown translocated to the nucleus in hippocampal neurons and stimulated transcription by phosphorylating CREB [56]. CAMK4 is predominantly localized in the nucleus and there is good evidence that it is responsible for Ca^2+^-dependent stimulation of transcription through phosphorylation of CREB at Ser133 [57]. Co-expression of CAMKK2 with CAMK4 resulted in a 14-fold enhancement of CREB-dependent gene expression compared to the solitary expression of CAMK4 only [58]. Thus, it is conceivable that CAMKK2-CAMK1/4-CREB signaling may be an alternative signaling pathway potentially regulating SDHs expression. However, validation of these hypotheses is subject to future studies.

A major finding in this study is the cell-type-specific PTMs of OXPHOS proteins including SDHs, specifically; SDHB under CAMKK2 deleted conditions which correlated with mitochondrial functional differences. The exact nature of the PTMs in two major SDHB pI fractions needs to be identified in the future. Phosphorylation usually induces an acidic shift in the pI [59] and single phosphorylation may alter pI by 1–2 pH units [60], therefore, multiple phosphorylations may be involved in shifting SDHB pI from basic (pH 9) to an acidic (pH 3–4) pI (Fig. 7EF). Interestingly, high throughput mass spectrometric analysis documented a variety of SDHB PTMs archived in the PhosphositePlus database [61, 62]. Mining of the publically available databases for the evidence of phosphorylation in the ETC components identified 284 reported phosphorylation events, includeing P-SDHA (S456, T24, and Y215/365604/606/629), P-SDHB (S222, T119, and Y61/216), P-UQCRC2 (S56/87/88/111/226/367, T86/100/113/369, and Y55/191/207), and P-ATP5A1 (S53/65/76/99/100/166/184/254/419/451, T225/264, and Y243/246/299/337/343/440) proteins [63]. Thus, it is tempting to suggest that CAMKK2 loss may have differentially altered the phosphorylation state of OXPHOS proteins, including SDHs and more specifically SDHB, and an altered phosphorylation state of the ETC machinery was responsible for the cell-type-specific differences in mitochondrial bioenergetics.

BN-PAGE is a convenient method to study OXPHOS-associated MPCs [45–47]. Our results indicated considerable cell-type-specific differences in the assembly of OXPHOS-associated MPCs under CAMKK2 deleted conditions which correlated with altered mitochondrial function. The current concept of mitochondrial architecture states that OXPHOS complexes are not randomly distributed within the inner mitochondrial membrane, but assemble into supramolecular structures [64–66]. Supercomplex formation is important for the stability of the ETC and for reducing the production of reactive oxygen species (ROS) [65, 67]. The majority of CI is found bound with a CIII dimer and CIV (CI + CIII_2_ + CIV), a structure that contains all complexes required to pass electrons from NADH to O_2_ and hence is known as a “respirasome” [65]. Other combinations also exist, for example, C-1 bound to a CIII dimer (CI + CIII_2_) [68], or CIII dimer bound to CIV (CIII_2_ + CIV_1_) independent of CI [64] or CII and CIV bound together to form a stand-alone megacomplex not associated with any of the other respiratory complexes [69]. The existence of a supercomplex/megacomplex-like structure is supported by the vertical alignment of OXPHOS complexes in a very high molecular weight (> 1200 kDa) and multiple 146–720 kDa complexes in HEK293 and HepG2 cells (Fig. 6C, G). The vertical alignment of SDHB and MT-CO2 in HEK293 and HepG2 cells is an indication of the existence of CII + CIV megacomplex (Fig. 6). Interestingly, increased levels of SDHB and an increased presence of CII + CIV megacomplex in CAMKK2^−/−^ HEK293 correlated to an enhanced CII-mediated state-3u respiration in the isolated mitochondria compared to parental mitochondria. Also, the reduced SDHB levels and disassembled CII + CIV megacomplex in CAMKK2^−/−^ HepG2 correlated to the decreased CII-mediated state-3u respiration in the isolated mitochondria compared to parental mitochondria. This supported the conclusion that CAMKK2 regulates mitochondrial respiration by influencing both OXPHOS assembly and abundance in a cell-type-specific manner. It has been suggested that the natural integrity of respirasome or megacomplexes can be partially compromised during BN-PAGE or cryo-EM sample preparation due to lack of natural environment, loss of cardiolipin, mitochondrial isolation (centrifugation and washing), solubilization (detergents), air–water interface, and random collision, which, therefore, may induce artifacts [70]. However, a comparison of natural or disintegrated super/megacomplexes between control and experimental sets under identical conditions may provide a clue to the structure–function relationship which is reflected in this study. Under CAMKK2 deleted conditions, OXPHOS-associated MPCs shifted to the relatively higher molecular weight regions in HEK293 (Fig. 6C) whereas, in HepG2 cells, the relative shift was comparatively towards the lower molecular weight regions (Fig. 6G). This cell-type-specific difference in the OXPHOS super/megacomplex profile correlated with an overall increased or decreased mitochondrial function, respectively.

One emerging question is – what is the relevance of an increase or decrease in uncoupled mitochondrial respiration? Uncoupling is a general term comprising diverse mechanisms [71]. In the coupled state, the proton motive force generated by electron transport is dissipated by the vectorial movement of protons across the inner mitochondrial membrane (IMM) through ATP synthase (CV), which generates energy in the form of ATP, thus proton flow is coupled with ATP production. In uncoupled respiration, the proton flow bypasses CV and is not coupled with ATP production. Uncoupled respiration occurs due to intrinsic “proton leaks” [72] or is mediated by uncoupling proteins (UCPs) or chemical mediators, for example, FCCP [73]. Uncoupling mechanisms involving UCPs generate a large amount of heat for thermoregulation, limit the production of ROS and assist in metabolic reprogramming [74]. FCCP-mediated uncoupling may involve some IMM proteins although the mechanistic details remain obscure [75]. In this context, it is important to note that STO-609 has been proposed as a mitochondrial uncoupler [76] and pharmacological inhibition of CAMKK2 in mice using STO-609 caused an acute increase in body temperature and a significant decrease in body weight. Furthermore, CAMKK2 downstream effector—AMPK has been linked to UCP2 for exerting a cardio-protective effect under mitochondrial dysfunction condition [77]. Thus, the cell-type-specific increased or decreased uncoupled respiration observed under CAMKK2 deleted condition supports previous reports and implies a role of CAMKK2 in thermoregulation and organ/tissue-specific metabolic reprogramming.

Another emerging question is—how does cellular respiration differ between unperturbed cells and isolated mitochondria and how does CAMKK2 regulate this process? It is difficult to provide a definitive explanation within the premise of this study, however, a reasonable hypothesis can be proposed based on the evidence in the existing literature. It is important to note that mitochondria are embedded in the cytosol of unperturbed cells, whereas, the cytosolic factors are absent in the isolated mitochondria. The latter may thus account for the difference in function. One cytosolic factor that regulates mitochondrial function is α-tubulin. Free dimeric α-tubulin mediates reversible blockage of VDAC, inhibiting VDAC permeability for ATP/ADP and other mitochondrial respiratory substrates, thus limiting mitochondrial function [78, 79]. Specific association of VDAC with α-tubulin was demonstrated in reconstituted planar lipid membranes [78, 79], as well as in immunoprecipitation studies [80]. VDAC is a component of MAM which creates an interface between the ER and mitochondria and provides a level of regulation in energy production and Ca^2+^ buffering [81]. It is important to note that the TEM-based experiment revealed that MAM is more frequent in HepG2 cells compared to HEK293 cells (Additional file 2: Fig. S2, Additional file 3: Fig. S3) which may account for some of the functional differences between these cell types. Since MAM structures were retained in the enriched ER/mitochondrial fraction (Additional file 3: Fig. S3), it is tempting to suggest that altered MPCs in the mitochondrial fraction may indicate potential impairment of MAM functioning under CAMKK2 deleted condition. Identification of the interacting proteins associated with OXPHOS and MAM under CAMKK2 deleted conditions may shed light on the discussed aspects of CAMKK2 regulation.

Another important question is what is the physiological relevance of CAMKK2-mediated differential regulation of mitochondrial metabolism through SDHs in relation to a specific cell type? This can be explained by discussing the role of CAMKK2 in the maintenance of whole-body energy homeostasis [2]. Organismal energy homeostasis is achieved by the communication between different metabolic organs and tissues to ensure balanced calorie intake, utilization, and proper energy flow. Some organs/tissues are destined for energy expenditure, for example, skeletal muscle, whereas others are dedicated to energy storage and balance, for example, adipose and liver tissues. Ca^2+^ is one of the most important second messengers [82] mediating a large variety of signal transduction pathways that regulate virtually all of the physiologic actions relevant to metabolism and organism function [2]. Therefore, it is conceivable that depending on the kinome, Ca^2+^/CAM-CAMKK2-downstream signaling will differ between cell types to meet the metabolic requirements of the organ/tissue. This has been reflected in our study as well as other studies involving tissue-specific diversity in CAMKK2 functioning [2]. For example, CAMKK2-AMPK signaling controls appetite and energy homeostasis in the brain [83], whereas, the CAMKK2-CAM4 axis contributes to nonalcoholic fatty liver disease (NAFLD) and is instrumental during the progression of hepatocellular carcinoma [84]. On the other hand, CAMKK2 plays a role in adaptive thermogenesis involving brown adipose tissue [2, 85]. In addition to Ca^2+^/CAM signaling, CAMKK2 is also regulated by two upstream Ser/Thr kinases, cyclin-dependent kinase 5 (CDK5) and glycogen synthase kinase 3 (GSK3); both phosphorylate CAMKK2 in the regulatory domain and control its autonomous activity [86, 87]. GSK3 is a downstream regulatory switch for numerous signaling pathways, including Wnt (Frizzled), Insulin (INSR), Reelin (VLDLR), Hedgehog (Patched), and GPCR signaling [88]. On the other hand, CDK5 is regulated by Calpain-dependent signaling [89]. Thus, CAMKK2 mediated cell-type-specific regulation of SDHs may indicate a mechanistic link wherein a multitude of signaling pathways converge to regulate mitochondrial function in a tissue/cell-type-specific manner to maintain energy homeostasis. This has been further reflected in the involvement of CAMKK2 in several metabolic diseases characterized by the manifestation of dysregulated mitochondrial function as part of their pathogenic process, including, for example, cancer [84, 90–92], obesity [83], diabetes [54], neurodegeneration [16, 17], and NAFLD [76]. One of the underlying pathogenic factors in these diseases is the excessive production of ROS through overactive OXPHOS which is damaging. It has been suggested that excessive mitochondrial ROS production in hepatocytes is one of the pathogenic factors for NAFLD [93]. In this context, CAMKK2-mediated downregulation of SDHB and reduced OXPHOS function in hepatocyte-like HepG2 cells [94] becomes physiologically relevant as it supports the findings that pharmacological treatment with STO-609, a selective small-molecule inhibitor of CAMKK2, conferred protection against NAFLD in a Streptozotocin and high fat-diet induced mouse model [76].

Another important physiological implication of this study involves the role of CAMKK2 in the regulation of inflammation [95]. It has been demonstrated that inhibition of CAMKK2 in myeloid cells suppresses tumor growth by increasing intratumoral accumulation of effector CD8 + T cells and immune-stimulatory myeloid subsets [91]. In this context, it is important to note that the TCA cycle intermediates succinate and fumarate are involved in “non-metabolic” signaling in both immunophysiology and disease contexts [96–98]. Succinate is considered pro-inflammatory [99, 100] and fumarate as an anti-inflammatory metabolite [98]. Succinate receptor 1 (SUCNR1) is highly expressed in dendritic cells resulting in the succinate-mediated production of pro-inflammatory cytokines and is responsible for enhancing activation of T helper cells and migration of dendritic cells, all underlying immunity [101, 102]. An elevated level of succinate in various diseases [96] is the therapeutic basis for targeting succinate metabolism [103–106]. On the other hand, fumarate signals through diverse signaling pathways regulating both innate and adaptive immune systems and rewires the epigenetic landscape of the cells through inhibition of histone and DNA demethylases [98]. Therefore, it is possible that reduction or loss of CAMKK2 function may alter SDH activity in a cell-type-specific manner, leading to a disturbed succinate/fumarate homeostasis in the tissue microenvironment which may have pro- or anti-inflammatory consequences depending on the context. Furthermore, impaired SDH activity may serve as the molecular basis for diverse signaling pathways associated with tumorigenesis which is discussed in detail by Moosavi et al. [107]. For example, increased succinate leads to the accumulation of hypoxia-inducible factor (HIF) which favors tumorigenesis by stimulating angiogenesis, reinforcing apoptosis resistance, and promoting the Warburg effect under hypoxia [107].

## Conclusions

This study provides novel insight into the cell-type-specificity and multiplicity of complex factors associated with CAMKK2-mediated regulation of mitochondrial function. These findings indicate that future studies should be directed towards understanding the mechanistic basis of calcium signaling and metabolic reprogramming, an area of research that has received minimal attention. Previously we established that CAMKK2-CAMK4 signaling regulates ER-mediated calcium homeostasis, receptor-mediated transferrin trafficking, and iron homeostasis [16, 17]. Therefore, the iron-sulfur protein SDHB representing a connecting link between the TCA cycle and ETS may serve as an important target for understanding the role of CAMKK2-CAMK4 signaling in calcium/iron homeostasis and metabolic regulation. The identification of cell-type-specific differences in CAMKK2 downstream effector kinase signaling and their effect on transcriptional, post-transcriptional, translational, and post-translational as well as structural assembly of OXPHOS proteins, specifically SDHs and more specifically SDHB, is the subject for future research to understand the mechanistic basis of CAMKK2 deficiency-induced phenotypes and its functional consequence in the pathogenesis of various diseases. Also, this study provides a hint to a unique therapeutic strategy in which manipulation of the yet uncharacterized cytosolic factor causing CAMKK2 mediated universal suppression of cellular respiration in different cell types may provide an opportunity to take the advantage of improved mitochondrial function under CAMKK2 deficient conditions in a cell/tissue/organ-specific context.

## Supplementary Information

**Additional file 1: Fig. S1.** Diagrammatic representation of the electron transport system showing different respiratory complexes, direction of electron flow, proton gradient, ATP production and inhibitors specific to different respiratory complexes.

**Additional file 2: Fig. S2.** Ultrastructure, distribution and organization of mitochondria in HEK293, and HepG2 cells. (A-B): TEM images of HEK293 (A), and HepG2 (B) cells grown on nitrocellulose membrane. N: nucleus, NU: nucleolus, M: mitochondria, RER: rough endoplasmic reticulum. The right panel images show a magnified view of the mitochondria and ER structures of the corresponding cell types. The red arrows indicate MAM (contact interface ER and mitochondria). The yellow arrow indicates sandwiched mitochondria between RERs and the cyan arrow indicates mitochondria half-encircled by RER..

**Additional file 3: Fig. S3.** Subcellular fractionation strategy. (A): Diagrammatic representation of the cell fractionation strategy. (B-C): TEM image of the HEK293 and HepG2-derived fractionated ER/mitochondrial pellet. Yellow rectangles represent enlarged view of the ER and mitochondrial structures presented in Fig. 3MN. (D): TEM image of the rough ER (RER) and mitochondria (M) in the enriched fraction. MAM: mitochondria associated ER membranes. The yellow arrow indicates cristae in the mitochondria.

**Additional file 4: Fig. S4.** Relative quantification of OXPHOS proteins and absolute quantification of SDHC mRNA levels in CAMKK2-defiicent cells. (A-D): Scatter plots showing relative amount of OXPHOS proteins in CAMKK2^−/−^ and parental (wild-type) HEK293 cells. Statistical significance from one-way ANOVA followed by multiple comparisons. (E–F): SDHB copy numbers in CAMKK2^−/−^ and parental HEK293 and HepG2 cells. Statistical analysis by t-test (unpaired), ns: p > 0.05.

**Additional file 5: Fig. S5.** Immunoblots showing relative amount of SDHs in ER/mitochondrial fractions from CAMKK2^−/−^ and parental HEK293 cells. (A) The immunoblots in A were generated by simultaneous use of mouse monoclonal anti-SDHA, -SDHB and -SDHC antibodies obtained from Santa Cruz Biotechnology (Table 1). NS: non-specific binding. (B): The immunoblot was generated using anti-OXPHOS antibody. The colored rectangles indicate relatively increased levels of the respective proteins.

**Additional file 6: Fig. S6.** Immunoblots showing relative amount of SDHs in ER/mitochondrial fractions from CAMKK2^−/−^ and parental HepG2 cells. The immunoblots in A-C were generated by co-immunoblotting using mouse monoclonal anti-SDHA, -SDHB and -SDHC antibodies obtained from Santa Cruz Biotechnology (Table 1). The SDHs antibodies were ineffective for co-immunoblotting using HepG2 cells. D: Oriole-stained total protein profile.

**Additional file 7: Fig. S7.** Cell-type-specific effect of CAMKK2 loss on the relative abundance of SDHA and SDHB in CII. (A-D): Immunoblots showing SDHA and SDHC-associated MPCs in the mitochondria of CAMKK2-deleted HEK293 and HepG2 cell clones and corresponding parental (wild-type) cells. The red and green rectangles: altered vertical alignment of SDHB and SDHC MPCs. The green connecting line indicates the direction of shift in the MPCs. Yellow arrows indicate blots incubated with a different antibody without stripping. (E–G): Immunoblots presented in A-D were false colored and overlaid to show the direction of relative shift for the individual proteins associated with CII. (H): Bar graphs showing the relative abundance of SDHB in the > 1200 kDa and 146–480 kDa SDHA-associated MPCs in the ER/mitochondrial fractions derived from parental and CAMKK2^−/−^ HEK293 and HepG2 cells. The SDHB percentage was calculated by first determining the total intensities of SDHA (X) and SDHB (Y) associated MPCs (> 1200 and 146–480 KDa) in the parental (X^WT^ and Y^WT^) and CAMKK2^−/−^ (X^KO^ and Y^KO^) cell types using the immunoblots captured under the same exposure time and Western blotting conditions. Subsequently, the relative abundance of SDHB in SDHA-associated MPCs was determined by using the formula: Parental (%) = [(X^WT^/Y^WT^)/(X^WT^/Y^WT^)]*100 and CAMKK2^−/−^ (%) = [(X^KO^/Y^KO^)/(X^WT^/Y^WT^)]*100. Data presented as Mean ± SEM, N = 3 replicates from three independent experiments. Statistical analysis by t-test (unpaired), * P ≤ 0.05.

**Additional file 8: Table S1.** Instrument running protocol.

## Data Availability

The datasets used and/or analyzed during the current study are available from the corresponding author on reasonable request.

## Abbreviations

AMPK: AMP-activated protein kinase
ASC: Ascorbate
ATP5A: ATP synthase subunit alpha 5
BN-PAGE: Blue-native polyacrylamide gel electrophoresis
C: Complex
CAMK1: Ca2+/CAM-dependent protein kinase I
CAMK4: Ca2+/CAM-dependent protein kinase 4
CAMKK2: Calcium (Ca^2+^)/calmodulin (CAM)-activated kinase kinase 2
CDK5: Cyclin-dependent kinase 5
CREB: cAMP response element-binding
ETC: Electron transport chain
ETS: Electron transport system
FCCP: 2-[2-[4-(Trifluoromethoxy)phenyl]hydrazinylidene]-propanedinitrile
Glu: Glucose
GSK3: Glycogen synthase kinase 3
HPA: Human Protein Atlas
IEF: Isoelectric focusing
IMM: Inner mitochondrial membrane
Mal: Malate
MAM: Mitochondrial-associated ER membranes
MSB: Mitochondrial stabilization buffer
MT-CO2: Mitochondrially Encoded Cytochrome C Oxidase II
NAFLD: Nonalcoholic fatty liver disease
NDUFB8: NADH-Ubiquinone Oxidoreductase Subunit B8
OCR: Oxygen consumption rate
Oligo: Oligomycin
OXPHOS: Oxidative phosphorylation
PBS: Phosphate-buffered saline
PTM: Post-translational modification
Pyr: Pyruvate
qRT-PCR: Quantitative real time-PCR
RCR: Respiratory control ratio
RER: Rough endoplasmic reticulum
ROS: Reactive oxygen species
Rtn/AA: Rotenone/antimycin-A
RT-PCR: Reverse-transcription polymerase chain reaction
SDH: Succinate dehydrogenase
SDHA: Succinate dehydrogenase subunit A
SDHB: Succinate dehydrogenase subunit B
SDHC: Succinate dehydrogenase subunit C
SIRT1: Sirtuin 1
Succ: Succinate
TEM: Transmission electron microscopy
TMPD: N,N,N9,N9-Tetramethylp-phenylenediamine
UQCRC2: Ubiquinol-Cytochrome C Reductase Core Protein 2
VDAC1: Voltage Dependent Anion Channel 1
