# Supplementary figures and images for: CAMKK2 regulates mitochondrial function by controlling succinate dehydrogenase expression, post-translational modification, megacomplex assembly, and activity in a cell-type-specific manner
Source: Cell Commun Signal. 2021 Sep 25;19:98. doi: 10.1186/s12964-021-00778-z (PMC8466908; doi:10.1186/s12964-021-00778-z)

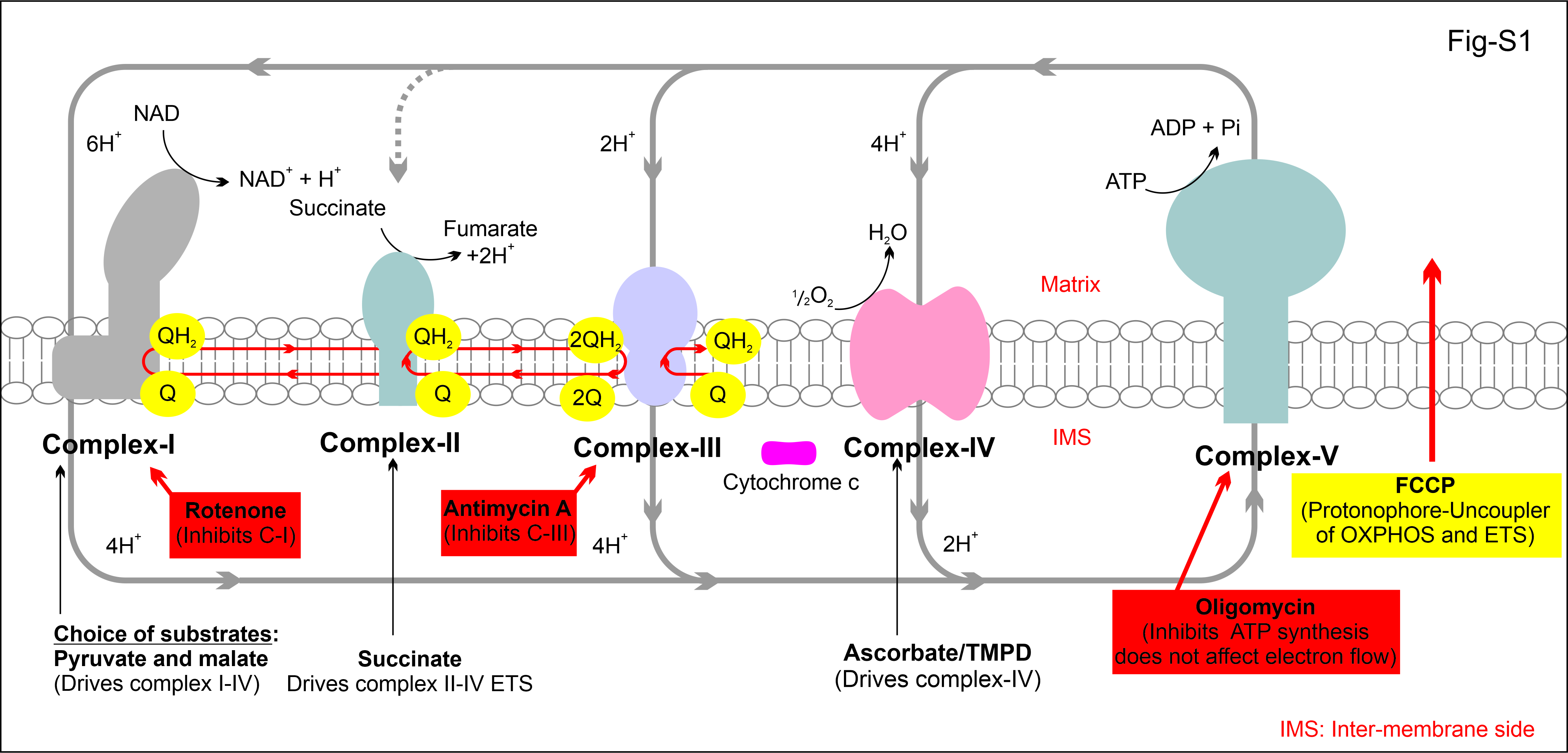

Supplement: Supplementary file 2 — Additional file 1: Fig. S1. Diagrammatic representation of the electron transport system showing different respiratory complexes, direction of electron flow, proton gradient, ATP production and inhibitors specific to different respiratory complexes. [file 12964_2021_778_MOESM2_ESM.jpg]

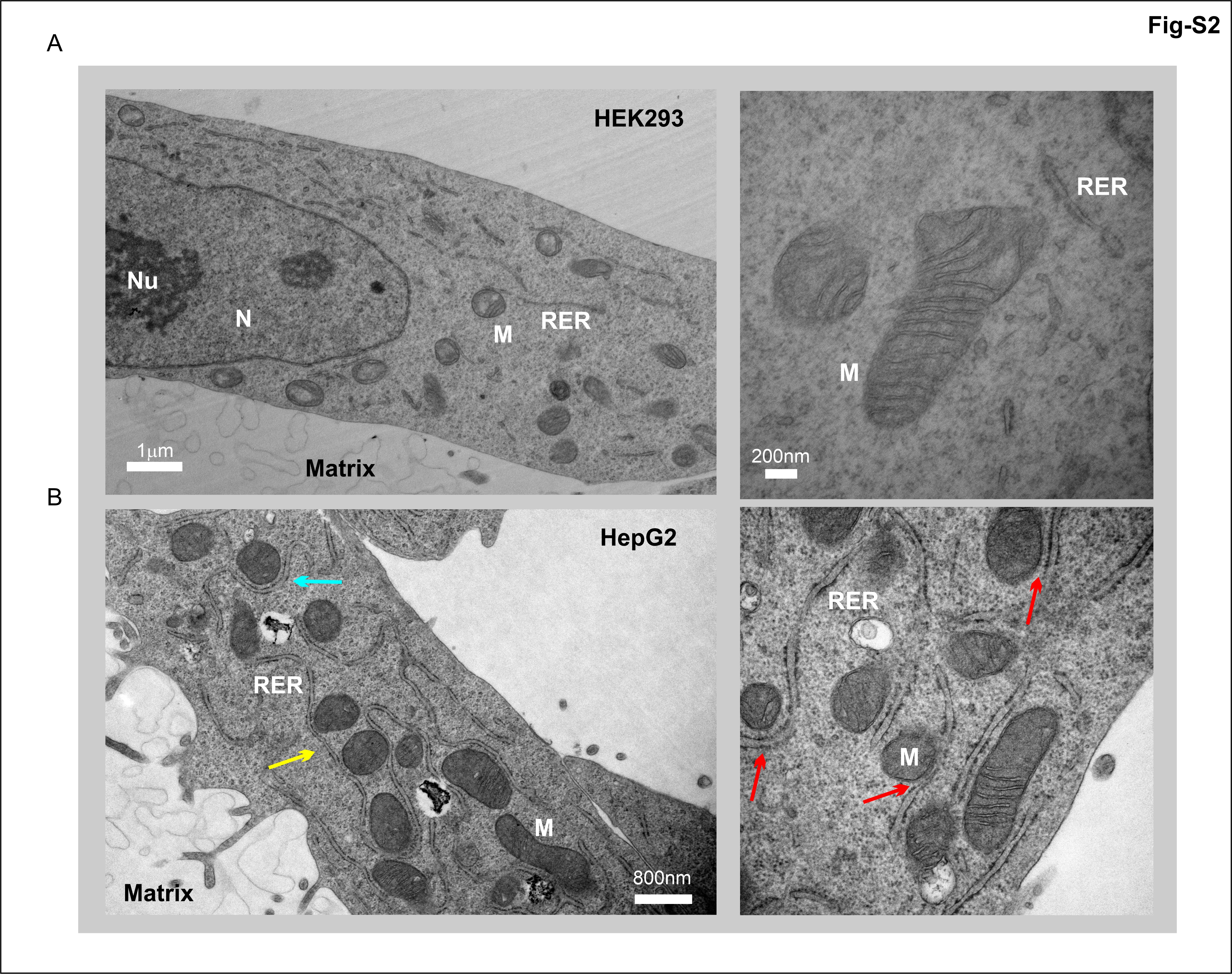

Supplement: Supplementary file 3 — Additional file 2: Fig. S2. Ultrastructure, distribution and organization of mitochondria in HEK293, and HepG2 cells. (A-B): TEM images of HEK293 (A), and HepG2 (B) cells grown on nitrocellulose membrane. N: nucleus, NU: nucleolus, M: mitochondria, RER: rough endoplasmic reticulum. The right panel images show a magnified view of the mitochondria and ER structures of the corresponding cell types. The red arrows indicate MAM (contact interface ER and mitochondria). The yellow arrow indicates sandwiched mitochondria between RERs and the cyan arrow indicates mitochondria half-encircled by RER.. [file 12964_2021_778_MOESM3_ESM.jpg]

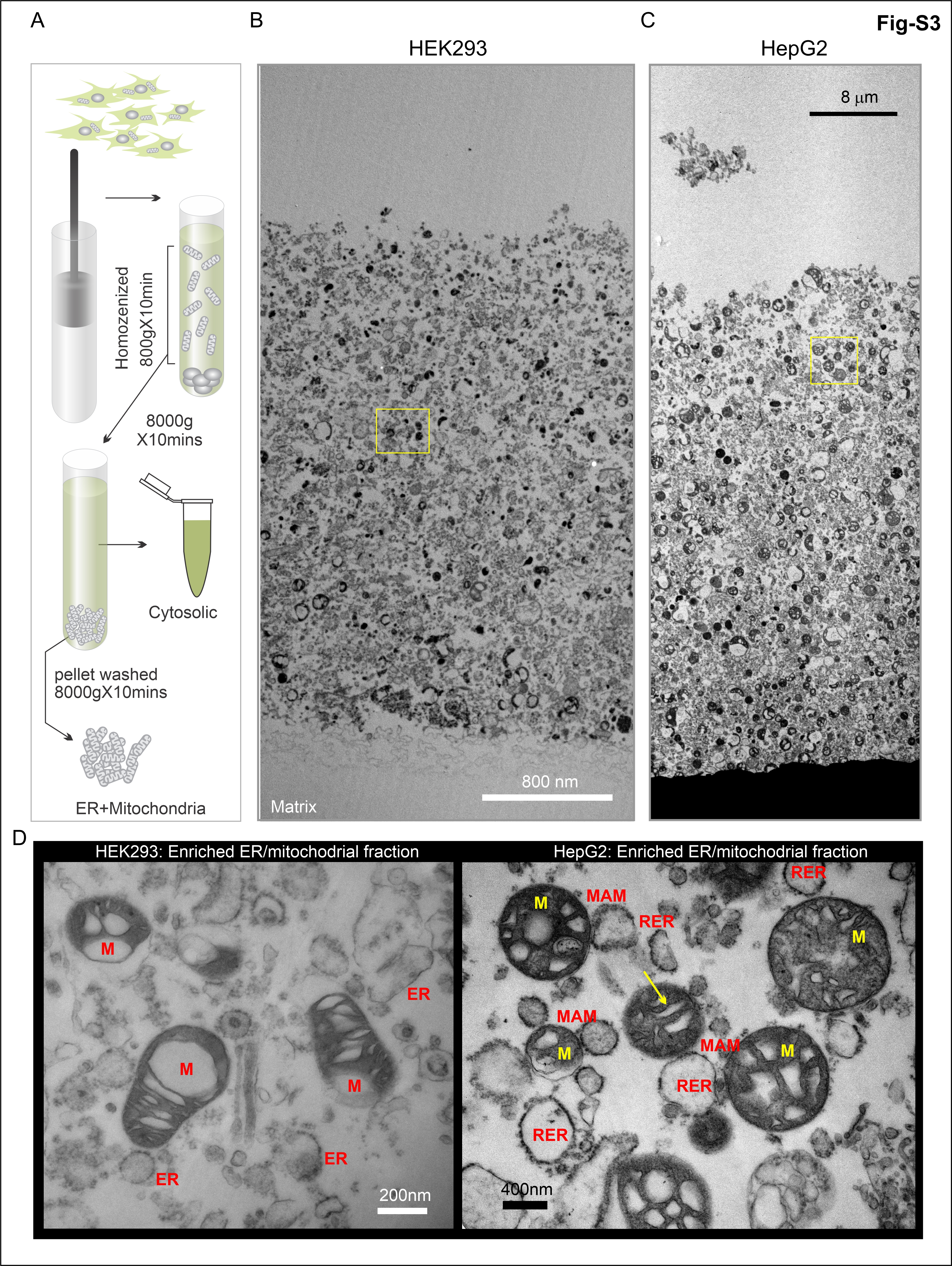

Supplement: Supplementary file 4 — Additional file 3: Fig. S3. Subcellular fractionation strategy. (A): Diagrammatic representation of the cell fractionation strategy. (B-C): TEM image of the HEK293 and HepG2-derived fractionated ER/mitochondrial pellet. Yellow rectangles represent enlarged view of the ER and mitochondrial structures presented in Fig. 3MN. (D): TEM image of the rough ER (RER) and mitochondria (M) in the enriched fraction. MAM: mitochondria associated ER membranes. The yellow arrow indicates cristae in the mitochondria. [file 12964_2021_778_MOESM4_ESM.jpg]

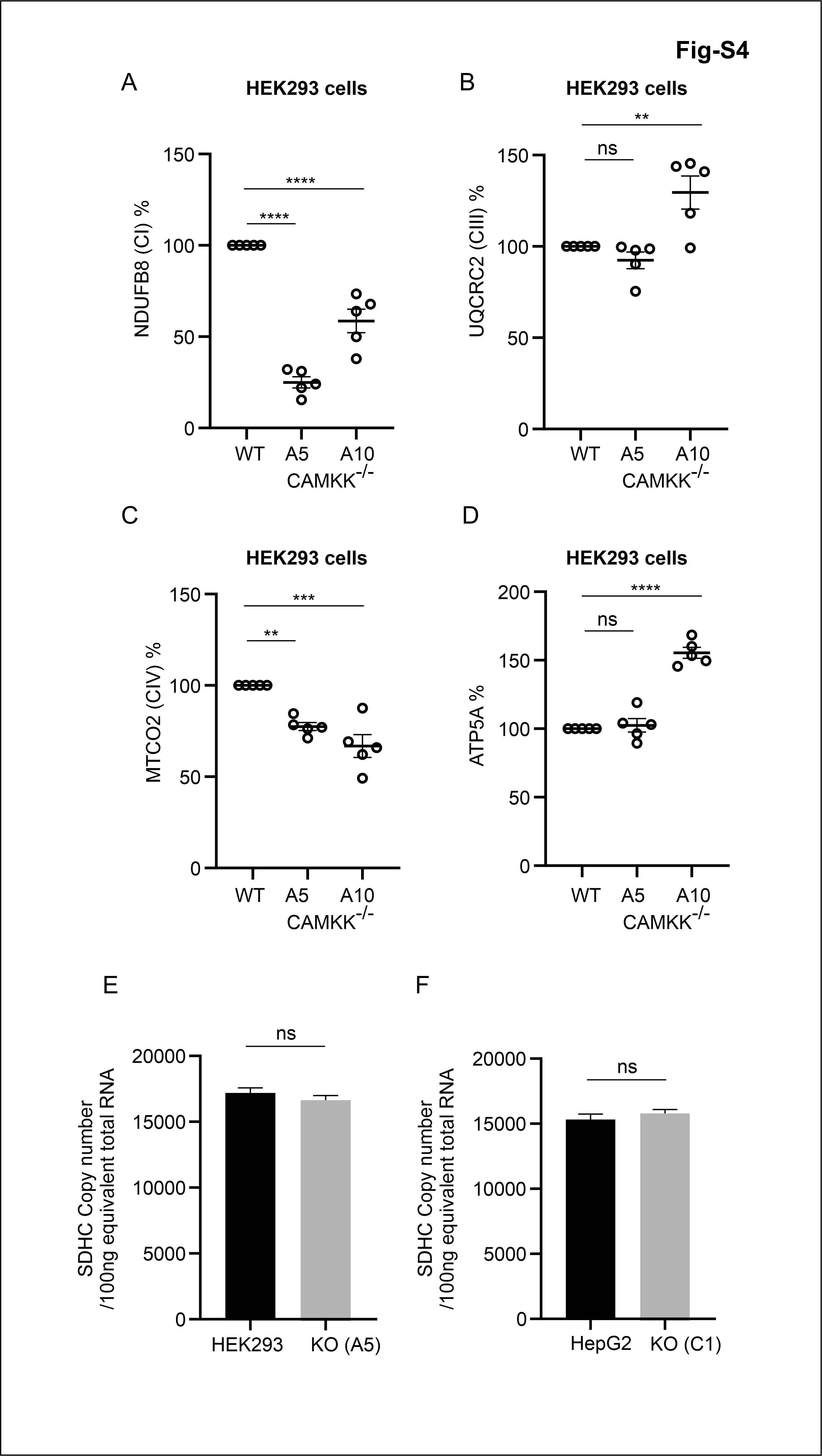

Supplement: Supplementary file 5 — Additional file 4: Fig. S4. Relative quantification of OXPHOS proteins and absolute quantification of SDHC mRNA levels in CAMKK2-defiicent cells. (A-D): Scatter plots showing relative amount of OXPHOS proteins in CAMKK2−/− and parental (wild-type) HEK293 cells. Statistical significance from one-way ANOVA followed by multiple comparisons. (E–F): SDHB copy numbers in CAMKK2−/− and parental HEK293 and HepG2 cells. Statistical analysis by t-test (unpaired), ns: p > 0.05. [file 12964_2021_778_MOESM5_ESM.jpg]

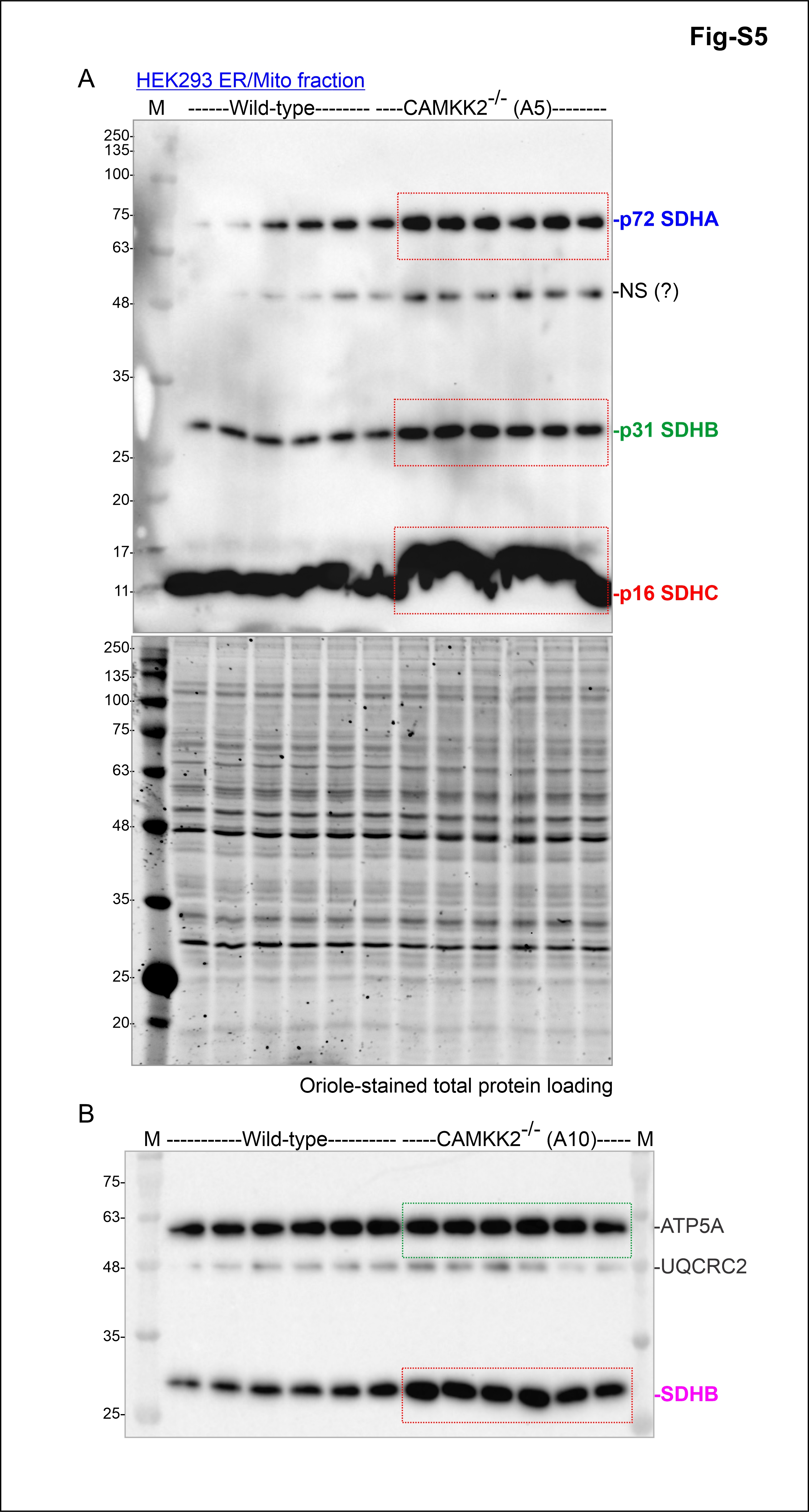

Supplement: Supplementary file 6 — Additional file 5: Fig. S5. Immunoblots showing relative amount of SDHs in ER/mitochondrial fractions from CAMKK2−/− and parental HEK293 cells. (A) The immunoblots in A were generated by simultaneous use of mouse monoclonal anti-SDHA, -SDHB and -SDHC antibodies obtained from Santa Cruz Biotechnology (Table 1). NS: non-specific binding. (B): The immunoblot was generated using anti-OXPHOS antibody. The colored rectangles indicate relatively increased levels of the respective proteins. [file 12964_2021_778_MOESM6_ESM.jpg]

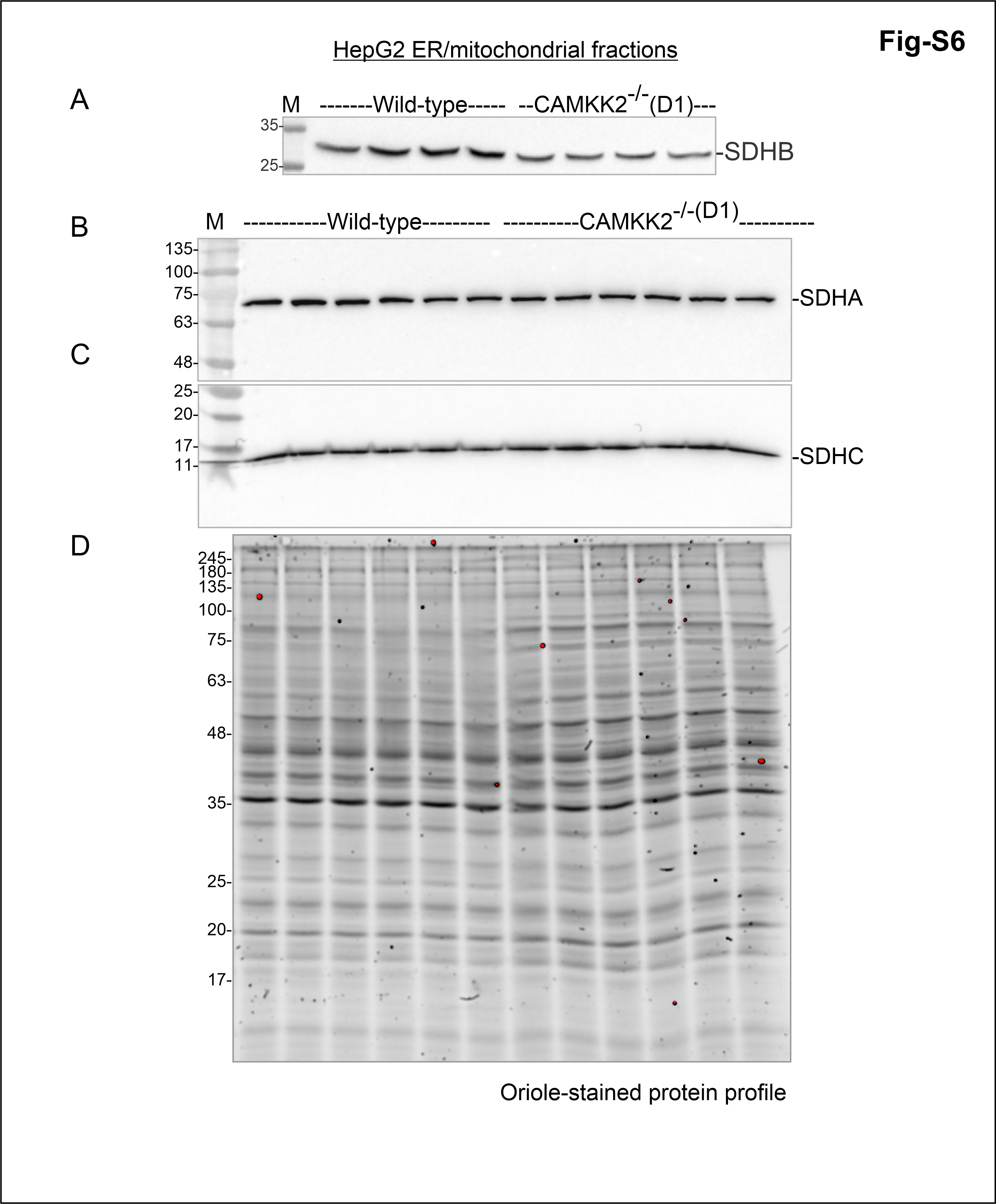

Supplement: Supplementary file 7 — Additional file 6: Fig. S6. Immunoblots showing relative amount of SDHs in ER/mitochondrial fractions from CAMKK2−/− and parental HepG2 cells. The immunoblots in A-C were generated by co-immunoblotting using mouse monoclonal anti-SDHA, -SDHB and -SDHC antibodies obtained from Santa Cruz Biotechnology (Table 1). The SDHs antibodies were ineffective for co-immunoblotting using HepG2 cells. D: Oriole-stained total protein profile. [file 12964_2021_778_MOESM7_ESM.jpg]

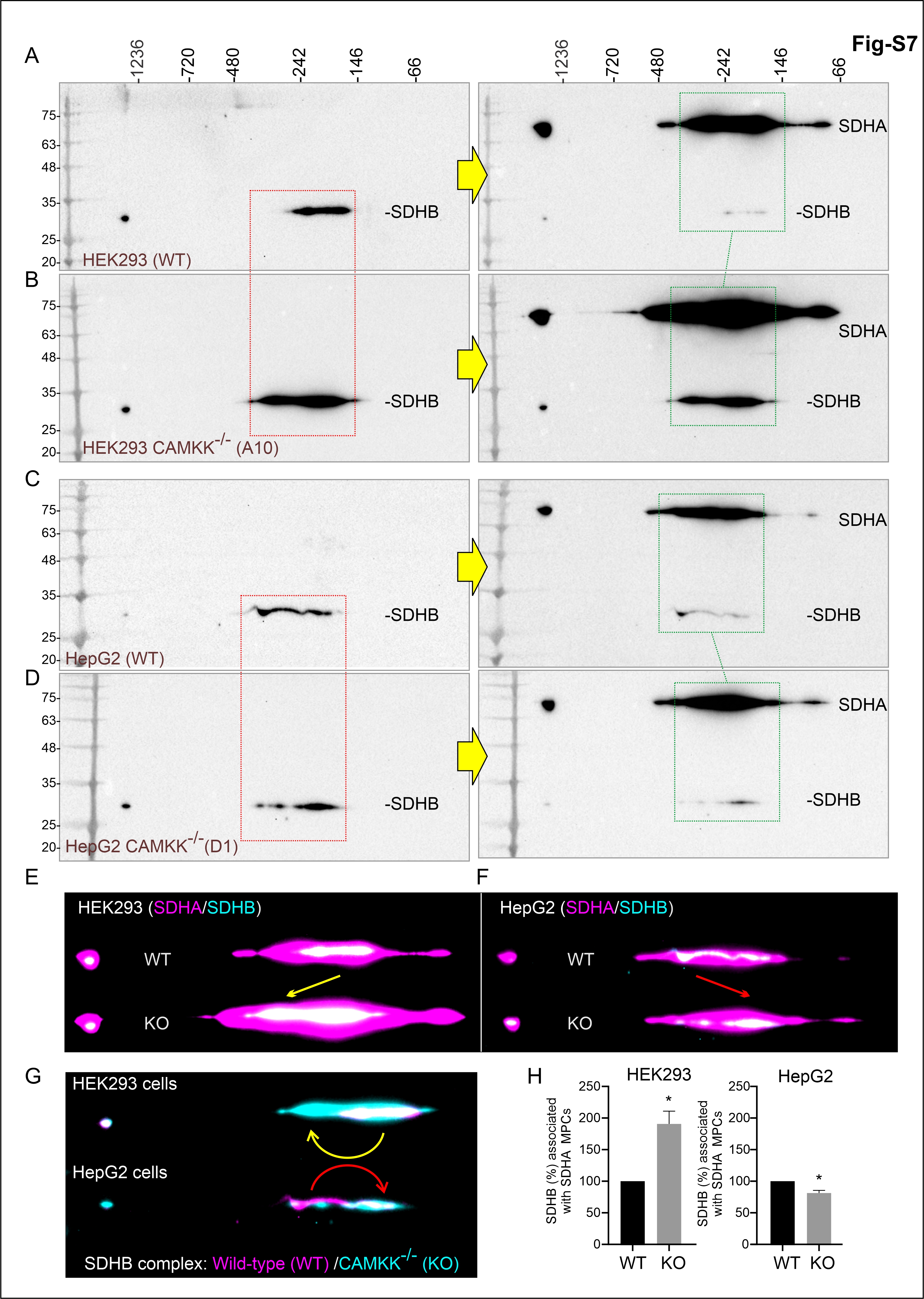

Supplement: Supplementary file 8 — Additional file 7: Fig. S7. Cell-type-specific effect of CAMKK2 loss on the relative abundance of SDHA and SDHB in CII. (A-D): Immunoblots showing SDHA and SDHC-associated MPCs in the mitochondria of CAMKK2-deleted HEK293 and HepG2 cell clones and corresponding parental (wild-type) cells. The red and green rectangles: altered vertical alignment of SDHB and SDHC MPCs. The green connecting line indicates the direction of shift in the MPCs. Yellow arrows indicate blots incubated with a different antibody without stripping. (E–G): Immunoblots presented in A-D were false colored and overlaid to show the direction of relative shift for the individual proteins associated with CII. (H): Bar graphs showing the relative abundance of SDHB in the > 1200 kDa and 146–480 kDa SDHA-associated MPCs in the ER/mitochondrial fractions derived from parental and CAMKK2−/− HEK293 and HepG2 cells. The SDHB percentage was calculated by first determining the total intensities of SDHA (X) and SDHB (Y) associated MPCs (> 1200 and 146–480 KDa) in the parental (XWT and YWT) and CAMKK2−/− (XKO and YKO) cell types using the immunoblots captured under the same exposure time and Western blotting conditions. Subsequently, the relative abundance of SDHB in SDHA-associated MPCs was determined by using the formula: Parental (%) = [(XWT/YWT)/(XWT/YWT)]*100 and CAMKK2−/− (%) = [(XKO/YKO)/(XWT/YWT)]*100. Data presented as Mean ± SEM, N = 3 replicates from three independent experiments. Statistical analysis by t-test (unpaired), * P ≤ 0.05. [file 12964_2021_778_MOESM8_ESM.jpg]
