# Supplementary material for: CAMKK2 regulates mitochondrial function by controlling succinate dehydrogenase expression, post-translational modification, megacomplex assembly, and activity in a cell-type-specific manner
Source: Cell Commun Signal. 2021 Sep 25;19:98. doi: 10.1186/s12964-021-00778-z (PMC8466908; doi:10.1186/s12964-021-00778-z)
Supplement: Supplementary file 9 — Additional file 8: Table S1. Instrument running protocol. [file 12964_2021_778_MOESM9_ESM.docx]

| Start protocol | | |
| --- | --- | --- |
| Command | Time (min) | Injection Port |
| Calibrate |  |  |
| Wait | 10 |  |
| Mix | 1 |  |
| Wait | 3 |  |
| Mix | 1 |  |
| Wait | 3 |  |
| Mix | 1 |  |
| Measure | 3 |  |
| Mix | 1 |  |
| Measure | 3 |  |
| Mix | 1 |  |
| Inject |  | A |
| Mix | 1 |  |
| Measure | 3 |  |
| Mix | 1 |  |
| Inject |  | B |
| Mix | 1 |  |
| Measure | 3 |  |
| Mix | 1 |  |
| Inject |  | C |
| Mix | 1 |  |
| Measure | 3 |  |
| Mix | 1 |  |
| Inject |  | D |
| Mix | 1 |  |
| Measure | 3 |  |
| Mix | 1 |  |

**Table-S1**: Instrument running protocol
